# Supplementary material for: ABIN1 is a negative regulator of effector functions in cytotoxic T cells
Source: EMBO Rep. 2024 Jun 14;25(8):17. doi: 10.1038/s44319-024-00179-6 (PMC11315980; doi:10.1038/s44319-024-00179-6)
Supplement: Supplementary file 1 — Appendix [file 44319_2024_179_MOESM1_ESM.pdf]

Additional supplementary material – Appendix

Table of content

Appendix Figure S1…………………………………………………………………………….. **Page 2**

Appendix Figure S2…………………………………………………………………………….. **Page 3**

Appendix Figure S3…………………………………………………………………………….. **Page 4**

Appendix Figure S4…………………………………………………………………………….. **Page 5**

Appendix Figure Legends……………………………………………………………………….. **Page 6**

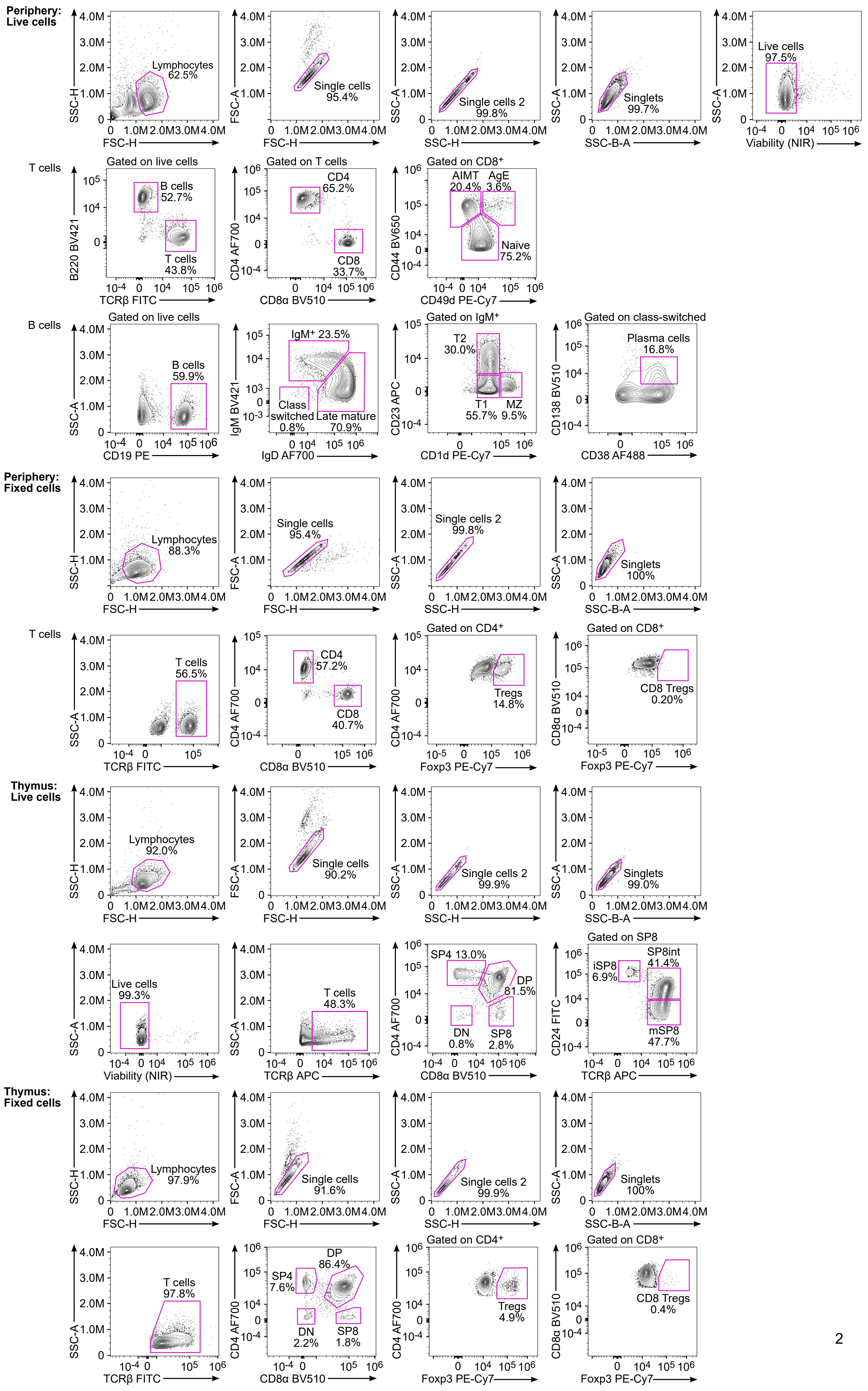
Periphery: Live cells
Lymphocytes 62.5%
Single cells 95.4%
Single cells 2 99.8%
Singlets 99.7%
Live cells 97.5%
SSC-H
FSC-H
FSC-A
SSC-A
SSC-B-A
Viability (NIR)
T cells
Gated on live cells
B cells 52.7%
T cells 43.8%
B220 BV421
TCRβ FITC
Gated on T cells
CD4 65.2%
CD8 33.7%
CD4 AF700
CD8α BV510
Gated on CD8+
AIMT 20.4%
AgE 3.6%
Naive 75.2%
CD44 BV650
CD49d PE-Cy7
B cells
Gated on live cells
B cells 59.9%
CD19 PE
IgM+ 23.5%
Class switched 0.8%
Late mature 70.9%
IgM BV421
IgD AF700
Gated on IgM+
T2 30.0%
T1 55.7%
MZ 9.5%
CD23 APC
CD1d PE-Cy7
Gated on class-switched
Plasma cells 16.8%
CD138 BV510
CD38 AF488
Periphery: Fixed cells
Lymphocytes 88.3%
Single cells 95.4%
Single cells 2 99.8%
Singlets 100%
T cells
T cells 56.5%
CD4 57.2%
CD8 40.7%
Gated on CD4+
Tregs 14.8%
Foxp3 PE-Cy7
Gated on CD8+
CD8 Tregs 0.20%
Thymus: Live cells
Lymphocytes 92.0%
Single cells 90.2%
Single cells 2 99.9%
Singlets 99.0%
Live cells 99.3%
T cells 48.3%
TCRβ APC
SP4 13.0%
DP 81.5%
DN 0.8%
SP8 2.8%
Gated on SP8
iSP8 6.9%
SP8int 41.4%
mSP8 47.7%
CD24 FITC
Thymus: Fixed cells
Lymphocytes 97.9%
Single cells 91.6%
Single cells 2 99.9%
Singlets 100%
T cells 97.8%
DP 86.4%
SP4 7.6%
DN 2.2%
SP8 1.8%
Gated on CD4+
Tregs 4.9%
Gated on CD8+
CD8 Tregs 0.4%

Appendix Figure S2

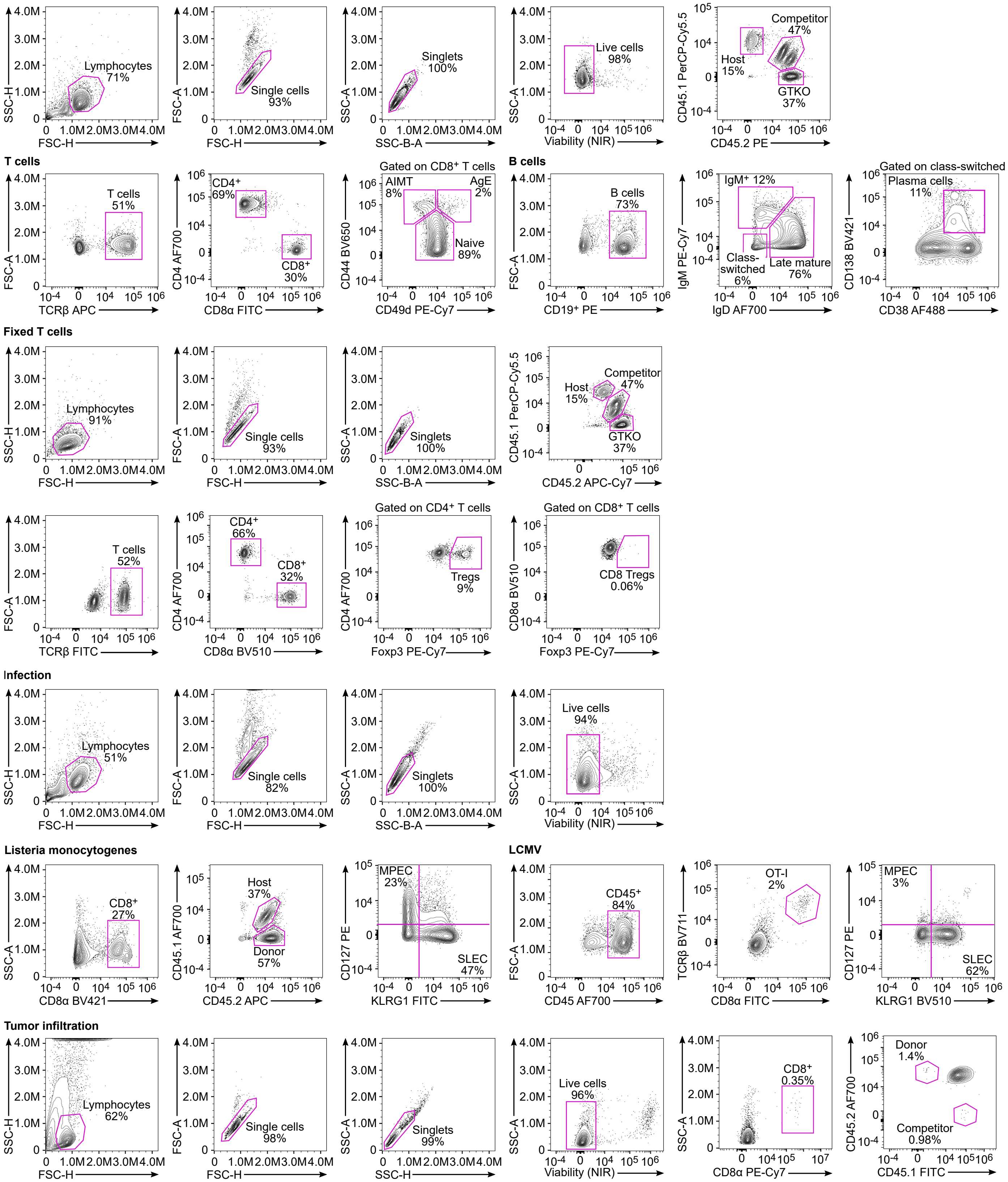

Immunophenotyping of *Abin1*$^{dE5/dE5}$ mice

Appendix Figure S3

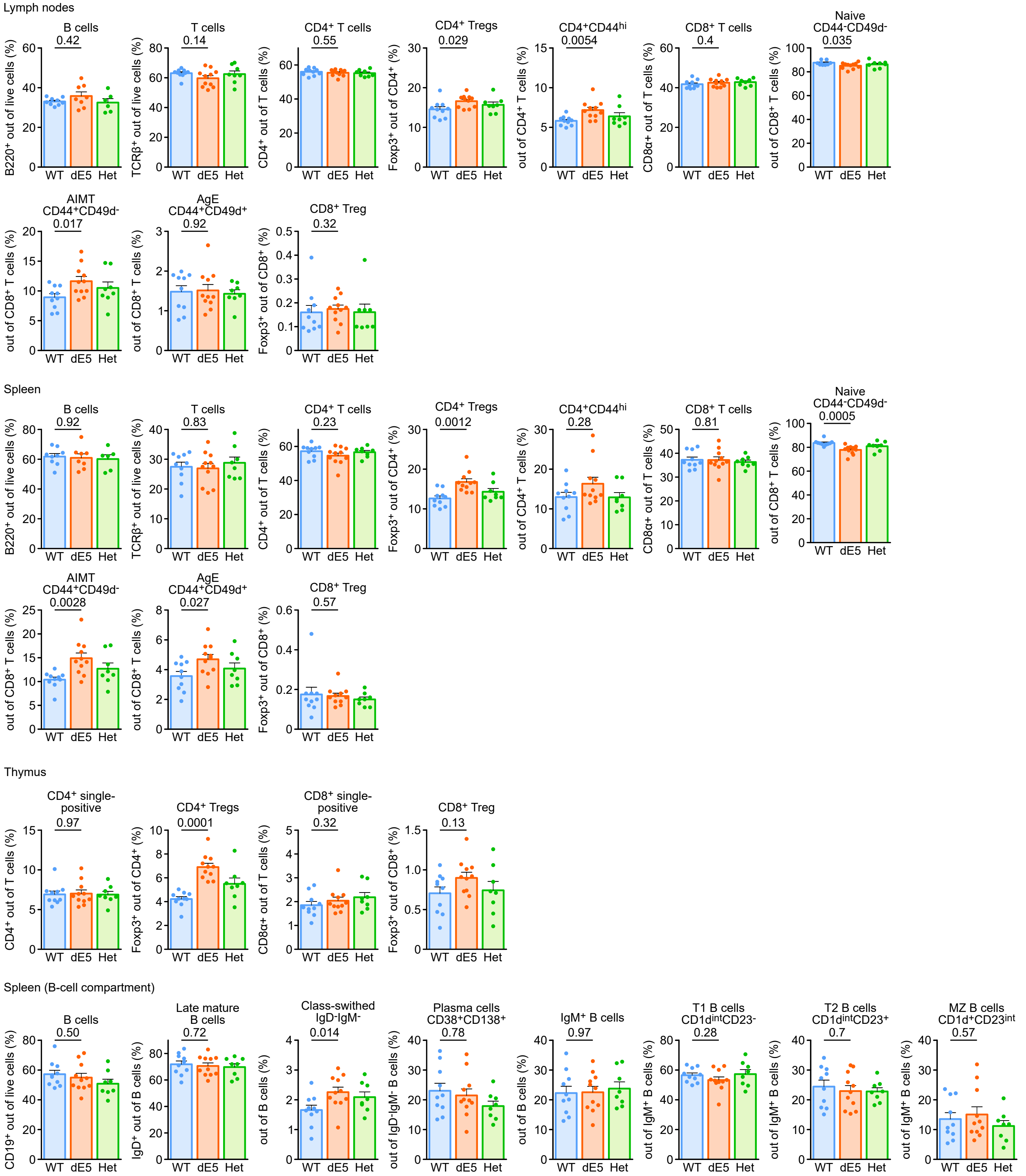

Appendix Figure S4

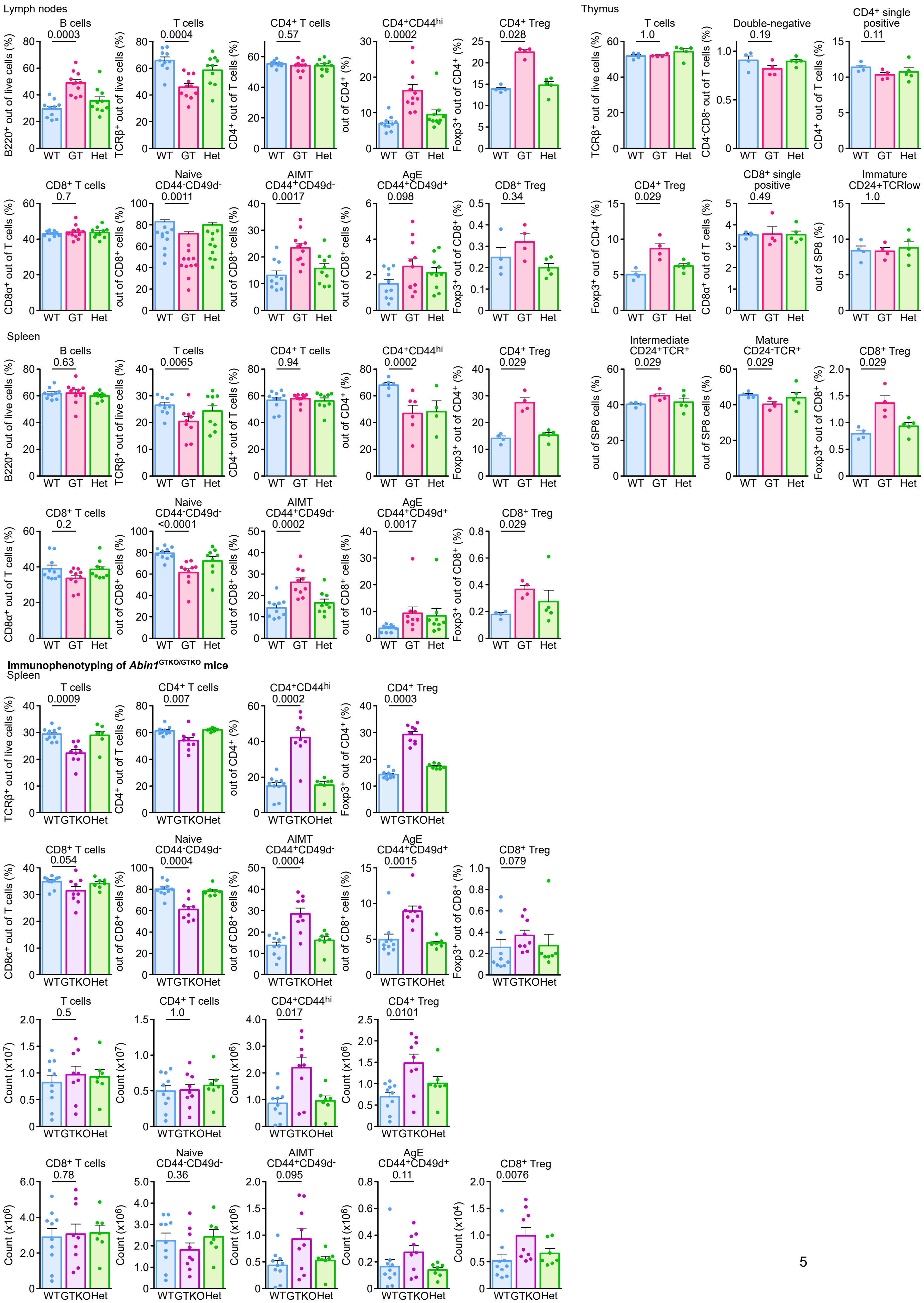

Appendix Figure Legends

Appendix Figure S1. Gating strategies for the analysis of live and fixed peripheral lymphocytes and thymocytes by flow cytometry used throughout this study.

Appendix Figure S2. Gating strategy used in analysis of bone marrow chimeras, infection (*Listeria* and LCMV), and cell infiltration in tumors by flow cytometry used in this study.

Appendix Figure S3. Immunophenotyping of $Abin1^{dE5/dE5}$ mice by flow cytometry. Quantification of indicated subsets are shown.

Data information: Data are presented as mean + SEM and p-values are indicated. Statistical significance was determined by two-tailed Mann-Whitney test.

Appendix Figure S4. Immunophenotyping of $Abin1^{GT/GT}$ and $Abin1^{GTKO/GTKO}$ mice by flow cytometry. Quantifications of indicated subsets are shown.

Data information: Data are presented as mean + SEM and p-values are indicated. Statistical significance was determined by two-tailed Mann-Whitney test.
