# Supplementary figures and images for: ABIN1 is a negative regulator of effector functions in cytotoxic T cells
Source: EMBO Rep. 2024 Jun 14;25(8):17. doi: 10.1038/s44319-024-00179-6 (PMC11315980; doi:10.1038/s44319-024-00179-6)

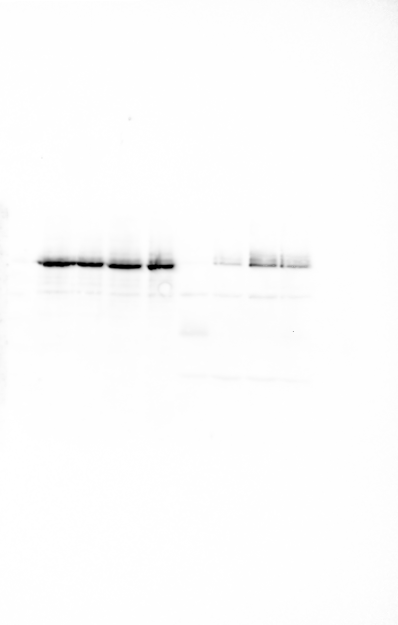

Supplement: Supplementary file 6 — Source data Fig. 1 [file 44319_2024_179_MOESM6_ESM.zip › Fig.1/Fig.1D/WB/A20.tif]

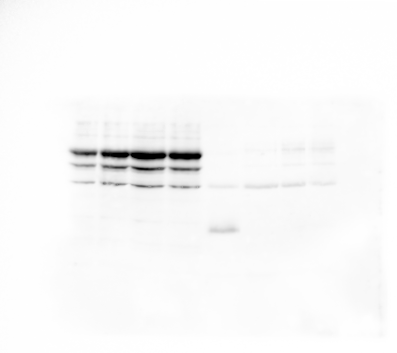

Supplement: Supplementary file 6 — Source data Fig. 1 [file 44319_2024_179_MOESM6_ESM.zip › Fig.1/Fig.1D/WB/ABIN1.tif]

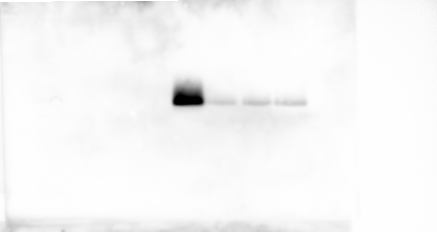

Supplement: Supplementary file 6 — Source data Fig. 1 [file 44319_2024_179_MOESM6_ESM.zip › Fig.1/Fig.1D/WB/FLAG.tif]

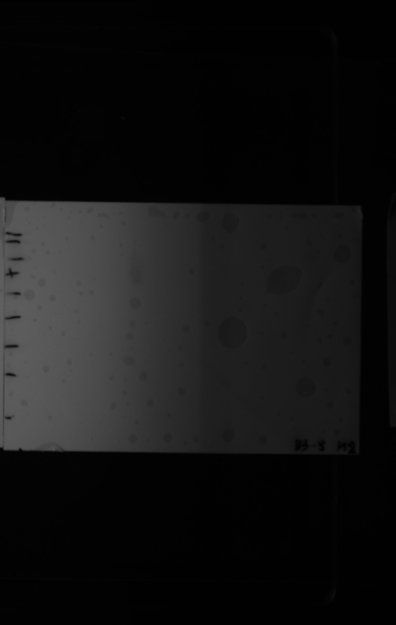

Supplement: Supplementary file 6 — Source data Fig. 1 [file 44319_2024_179_MOESM6_ESM.zip › Fig.1/Fig.1D/WB/marker A20.tif]

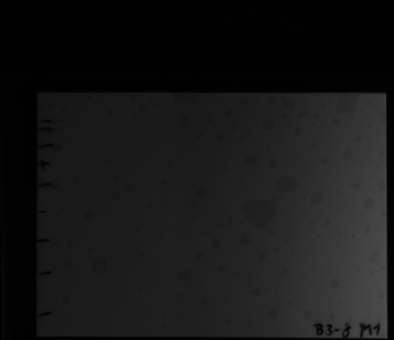

Supplement: Supplementary file 6 — Source data Fig. 1 [file 44319_2024_179_MOESM6_ESM.zip › Fig.1/Fig.1D/WB/marker ABIN1.tif]

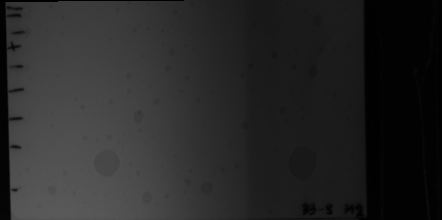

Supplement: Supplementary file 6 — Source data Fig. 1 [file 44319_2024_179_MOESM6_ESM.zip › Fig.1/Fig.1D/WB/marker FLAG.tif]

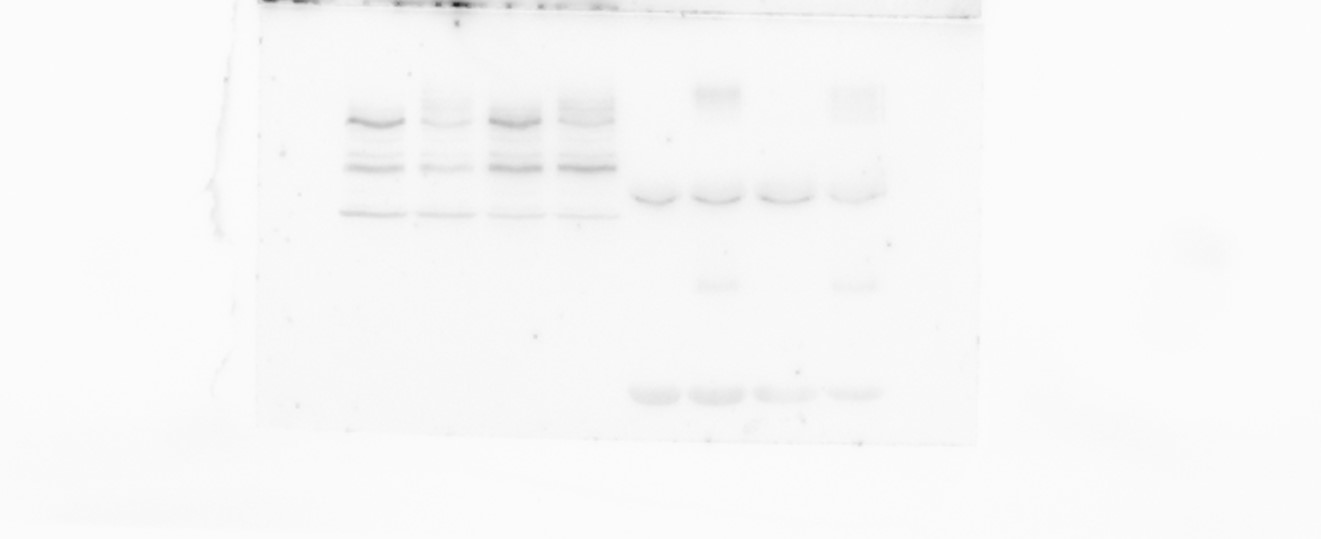

Supplement: Supplementary file 6 — Source data Fig. 1 [file 44319_2024_179_MOESM6_ESM.zip › Fig.1/Fig.1E/WB/A20.jpg]

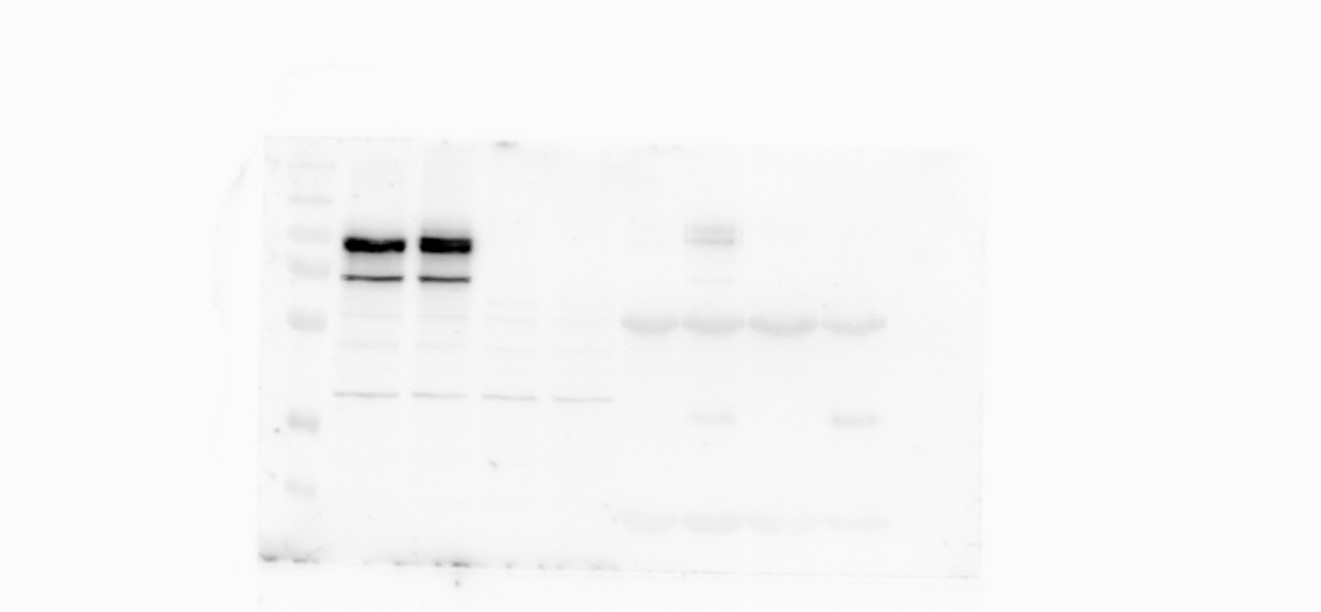

Supplement: Supplementary file 6 — Source data Fig. 1 [file 44319_2024_179_MOESM6_ESM.zip › Fig.1/Fig.1E/WB/ABIN1.jpg]

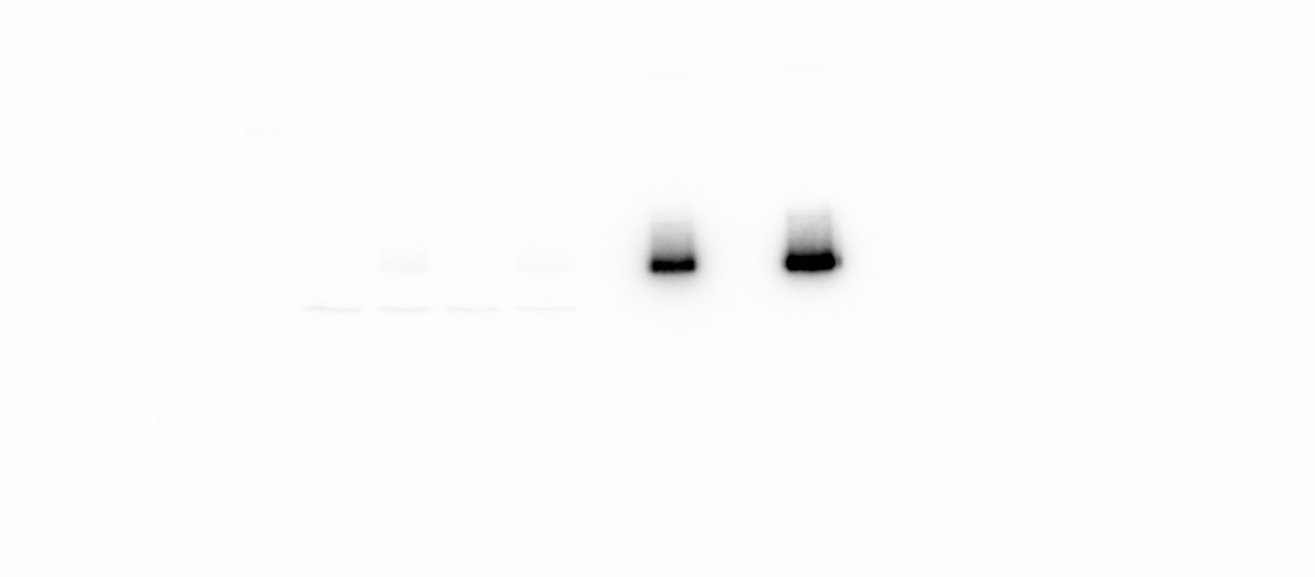

Supplement: Supplementary file 6 — Source data Fig. 1 [file 44319_2024_179_MOESM6_ESM.zip › Fig.1/Fig.1E/WB/FLAG.tif]

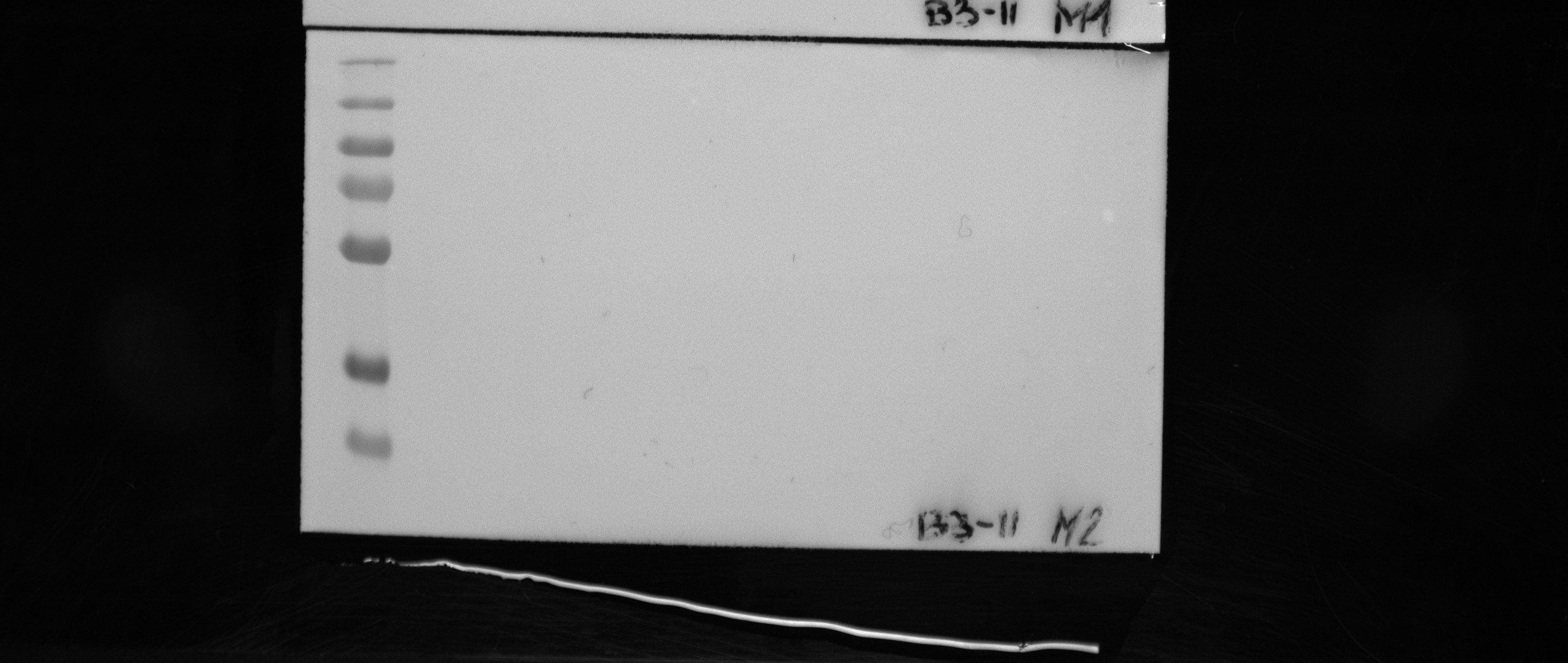

Supplement: Supplementary file 6 — Source data Fig. 1 [file 44319_2024_179_MOESM6_ESM.zip › Fig.1/Fig.1E/WB/marker A20.jpg]

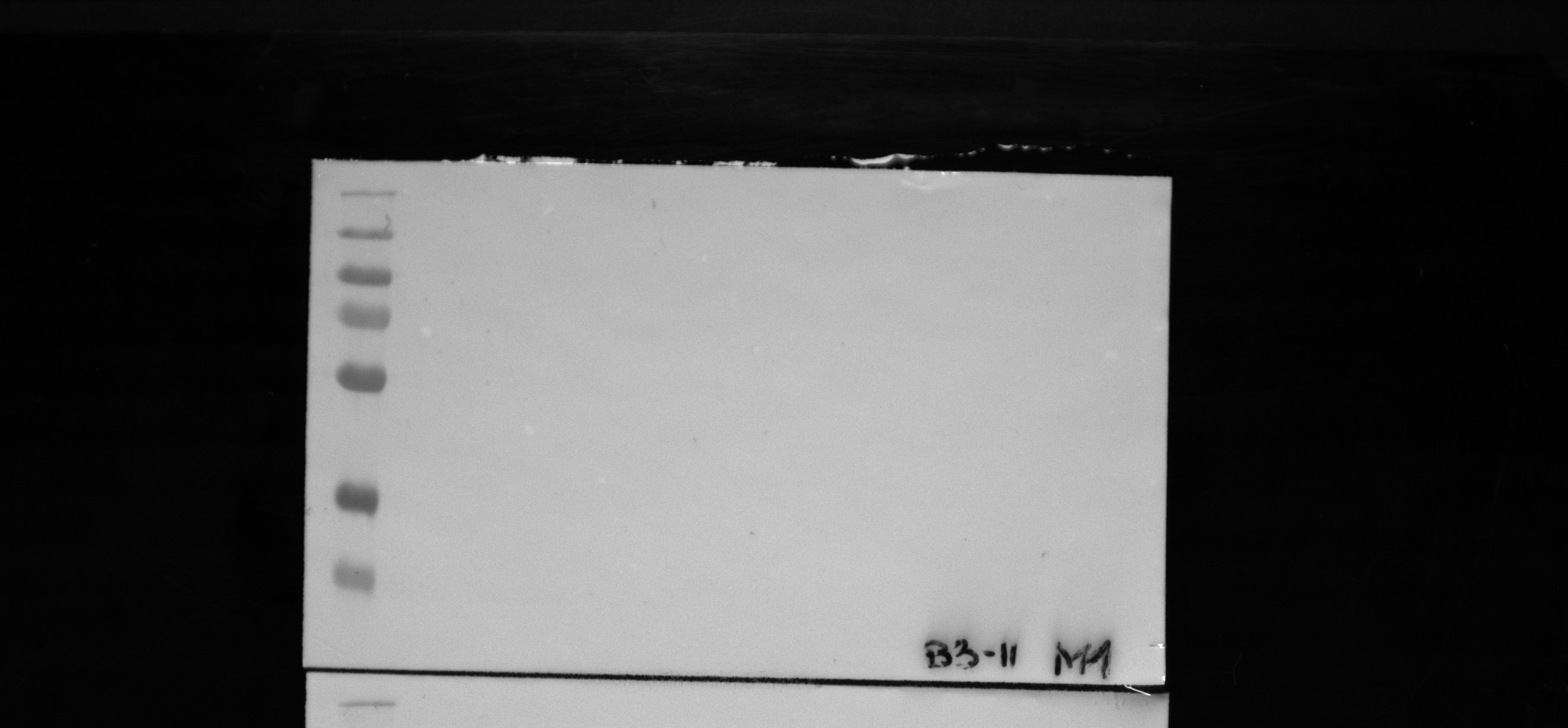

Supplement: Supplementary file 6 — Source data Fig. 1 [file 44319_2024_179_MOESM6_ESM.zip › Fig.1/Fig.1E/WB/marker ABIN1.jpg]

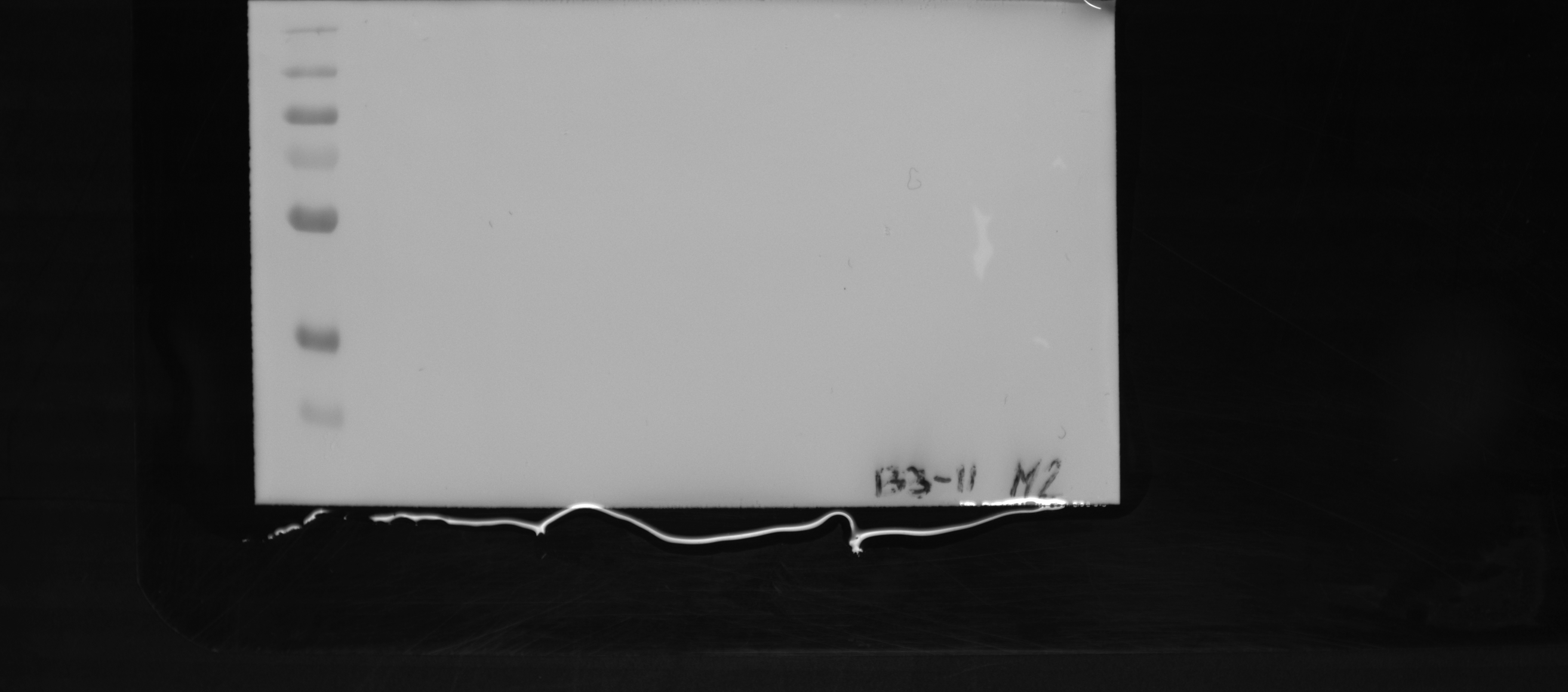

Supplement: Supplementary file 6 — Source data Fig. 1 [file 44319_2024_179_MOESM6_ESM.zip › Fig.1/Fig.1E/WB/marker FLAG.tif]

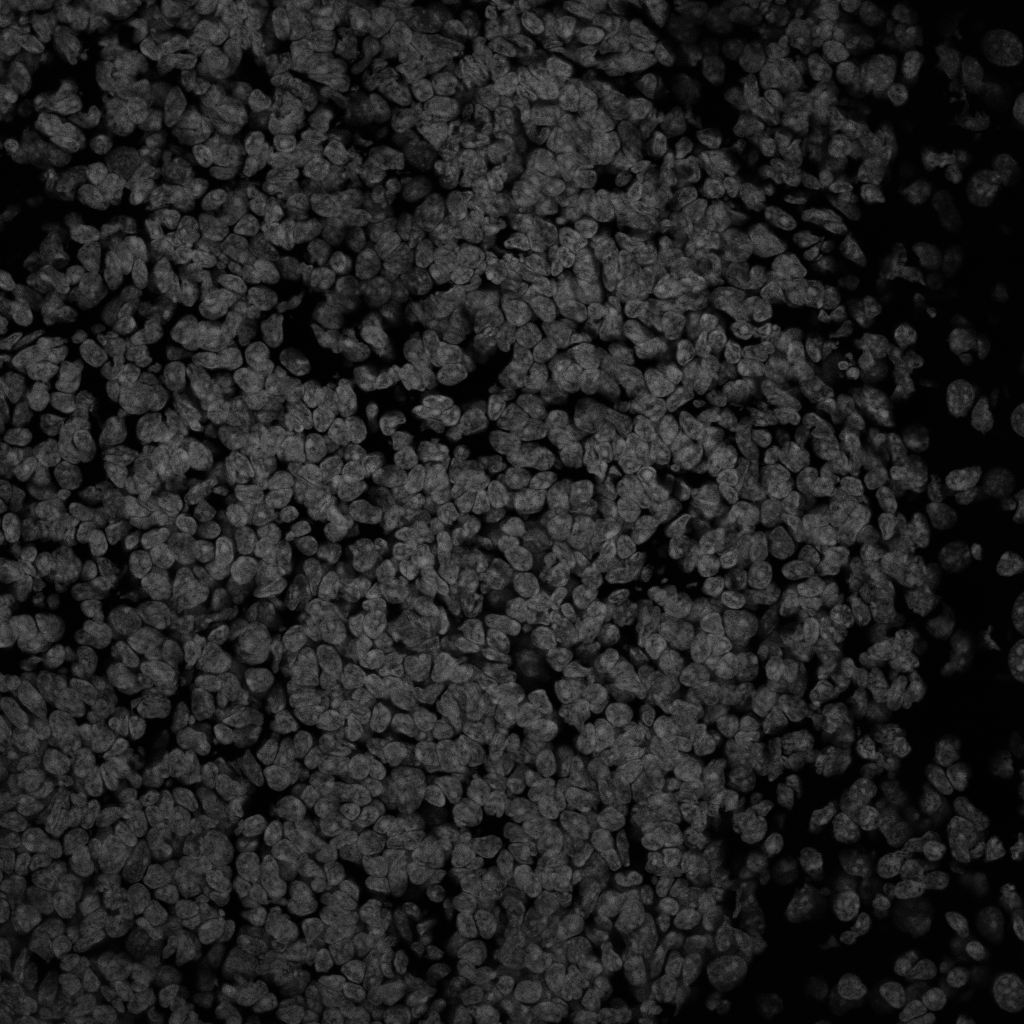

Supplement: Supplementary file 7 — Source data Fig. 2 [file 44319_2024_179_MOESM7_ESM.zip › Fig.2/Fig.2D/Microscopy/GTKO_DAPI-CD45-2-AF488-CD8-AF555-CD4-AF647.tif]

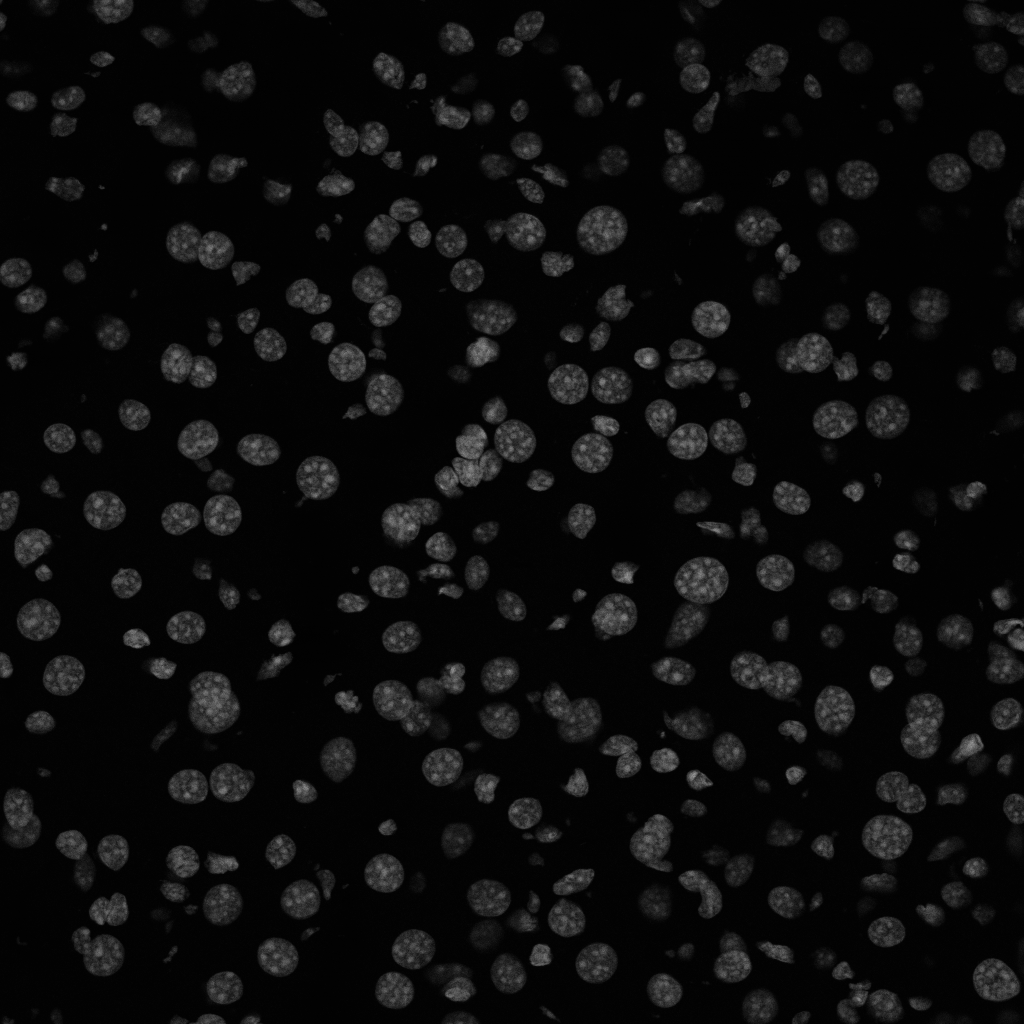

Supplement: Supplementary file 7 — Source data Fig. 2 [file 44319_2024_179_MOESM7_ESM.zip › Fig.2/Fig.2D/Microscopy/WT_DAPI-CD45-2-AF488-CD8-AF555-CD4-AF647.tif]

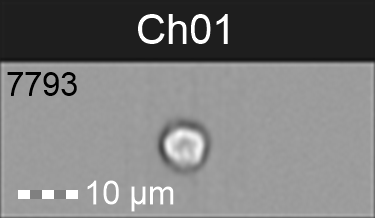

Supplement: Supplementary file 10 — Source data Fig. 5 [file 44319_2024_179_MOESM10_ESM.zip › Fig.5/Fig.5C/Microscopy/NFAT/GTKO 0.1uM OVA_BF.png]

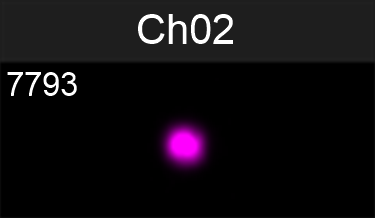

Supplement: Supplementary file 10 — Source data Fig. 5 [file 44319_2024_179_MOESM10_ESM.zip › Fig.5/Fig.5C/Microscopy/NFAT/GTKO 0.1uM OVA_Ch02.png]

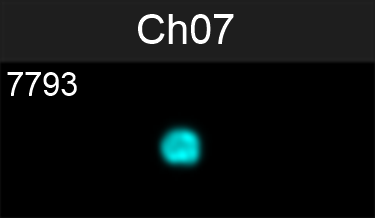

Supplement: Supplementary file 10 — Source data Fig. 5 [file 44319_2024_179_MOESM10_ESM.zip › Fig.5/Fig.5C/Microscopy/NFAT/GTKO 0.1uM OVA_Ch07.png]

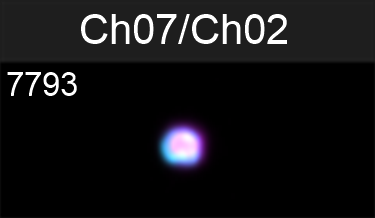

Supplement: Supplementary file 10 — Source data Fig. 5 [file 44319_2024_179_MOESM10_ESM.zip › Fig.5/Fig.5C/Microscopy/NFAT/GTKO 0.1uM OVA_overlay.png]

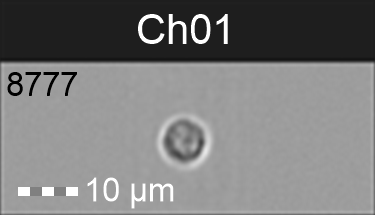

Supplement: Supplementary file 10 — Source data Fig. 5 [file 44319_2024_179_MOESM10_ESM.zip › Fig.5/Fig.5C/Microscopy/NFAT/GTKO 1uM OVA_BF.png]

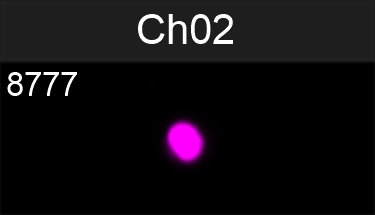

Supplement: Supplementary file 10 — Source data Fig. 5 [file 44319_2024_179_MOESM10_ESM.zip › Fig.5/Fig.5C/Microscopy/NFAT/GTKO 1uM OVA_Ch02.png]

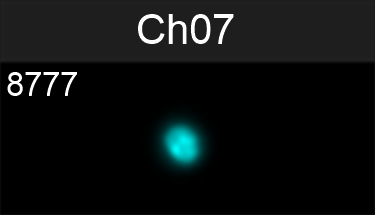

Supplement: Supplementary file 10 — Source data Fig. 5 [file 44319_2024_179_MOESM10_ESM.zip › Fig.5/Fig.5C/Microscopy/NFAT/GTKO 1uM OVA_Ch07.png]

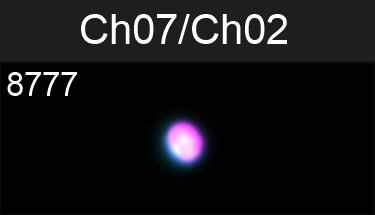

Supplement: Supplementary file 10 — Source data Fig. 5 [file 44319_2024_179_MOESM10_ESM.zip › Fig.5/Fig.5C/Microscopy/NFAT/GTKO 1uM OVA_overlay.png]

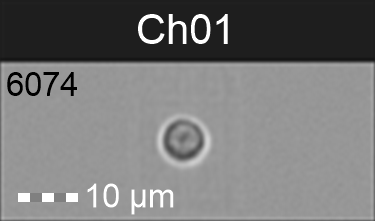

Supplement: Supplementary file 10 — Source data Fig. 5 [file 44319_2024_179_MOESM10_ESM.zip › Fig.5/Fig.5C/Microscopy/NFAT/GTKO no OVA_BF.png]

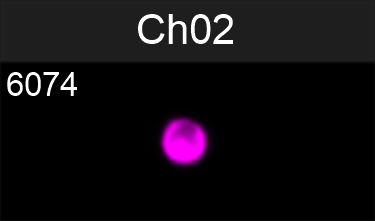

Supplement: Supplementary file 10 — Source data Fig. 5 [file 44319_2024_179_MOESM10_ESM.zip › Fig.5/Fig.5C/Microscopy/NFAT/GTKO no OVA_Ch02.png]

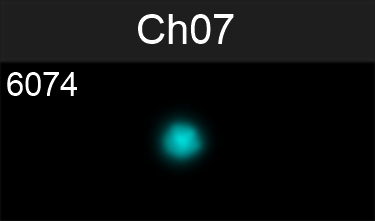

Supplement: Supplementary file 10 — Source data Fig. 5 [file 44319_2024_179_MOESM10_ESM.zip › Fig.5/Fig.5C/Microscopy/NFAT/GTKO no OVA_Ch07.png]

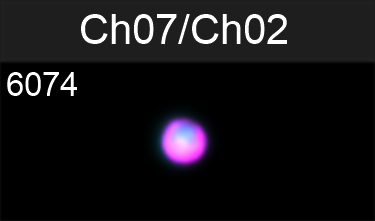

Supplement: Supplementary file 10 — Source data Fig. 5 [file 44319_2024_179_MOESM10_ESM.zip › Fig.5/Fig.5C/Microscopy/NFAT/GTKO no OVA_overlay.png]

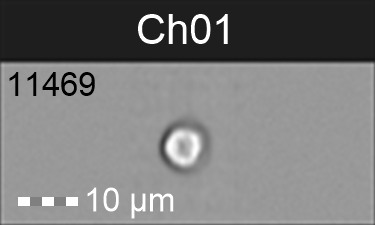

Supplement: Supplementary file 10 — Source data Fig. 5 [file 44319_2024_179_MOESM10_ESM.zip › Fig.5/Fig.5C/Microscopy/NFAT/WT 0.1uM OVA_BF.png]

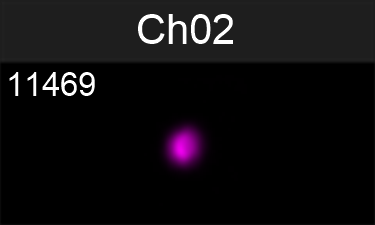

Supplement: Supplementary file 10 — Source data Fig. 5 [file 44319_2024_179_MOESM10_ESM.zip › Fig.5/Fig.5C/Microscopy/NFAT/WT 0.1uM OVA_Ch02.png]

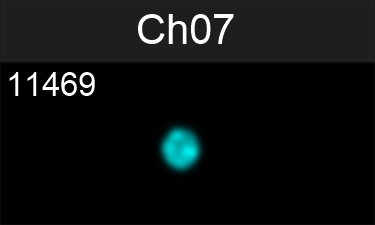

Supplement: Supplementary file 10 — Source data Fig. 5 [file 44319_2024_179_MOESM10_ESM.zip › Fig.5/Fig.5C/Microscopy/NFAT/WT 0.1uM OVA_Ch07.png]

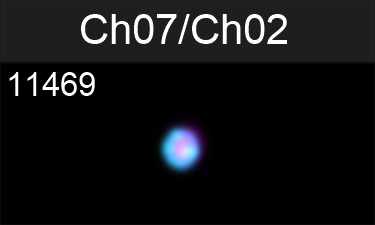

Supplement: Supplementary file 10 — Source data Fig. 5 [file 44319_2024_179_MOESM10_ESM.zip › Fig.5/Fig.5C/Microscopy/NFAT/WT 0.1uM OVA_overlay.png]

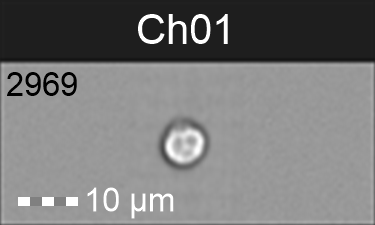

Supplement: Supplementary file 10 — Source data Fig. 5 [file 44319_2024_179_MOESM10_ESM.zip › Fig.5/Fig.5C/Microscopy/NFAT/WT 1uM OVA_BF.png]

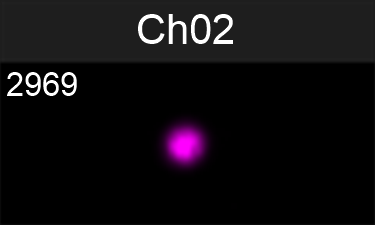

Supplement: Supplementary file 10 — Source data Fig. 5 [file 44319_2024_179_MOESM10_ESM.zip › Fig.5/Fig.5C/Microscopy/NFAT/WT 1uM OVA_Ch02.png]

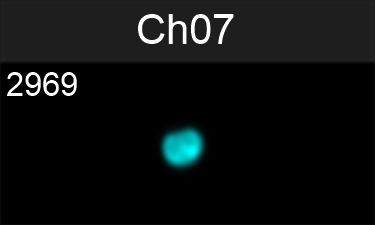

Supplement: Supplementary file 10 — Source data Fig. 5 [file 44319_2024_179_MOESM10_ESM.zip › Fig.5/Fig.5C/Microscopy/NFAT/WT 1uM OVA_Ch07.png]

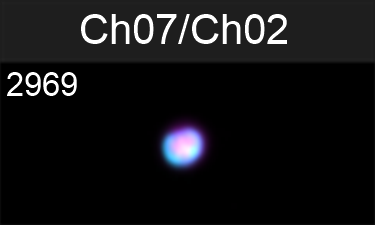

Supplement: Supplementary file 10 — Source data Fig. 5 [file 44319_2024_179_MOESM10_ESM.zip › Fig.5/Fig.5C/Microscopy/NFAT/WT 1uM OVA_overlay.png]

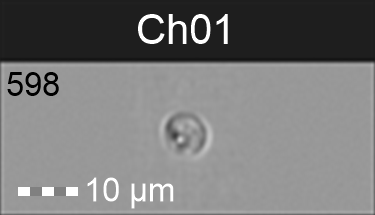

Supplement: Supplementary file 10 — Source data Fig. 5 [file 44319_2024_179_MOESM10_ESM.zip › Fig.5/Fig.5C/Microscopy/NFAT/WT no OVA_BF.png]

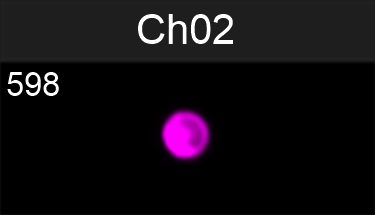

Supplement: Supplementary file 10 — Source data Fig. 5 [file 44319_2024_179_MOESM10_ESM.zip › Fig.5/Fig.5C/Microscopy/NFAT/WT no OVA_Ch02.png]

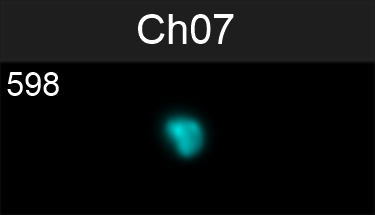

Supplement: Supplementary file 10 — Source data Fig. 5 [file 44319_2024_179_MOESM10_ESM.zip › Fig.5/Fig.5C/Microscopy/NFAT/WT no OVA_Ch07.png]

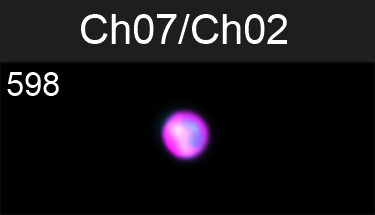

Supplement: Supplementary file 10 — Source data Fig. 5 [file 44319_2024_179_MOESM10_ESM.zip › Fig.5/Fig.5C/Microscopy/NFAT/WT no OVA_overlay.png]

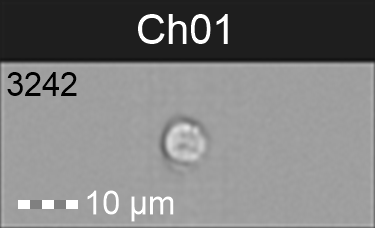

Supplement: Supplementary file 10 — Source data Fig. 5 [file 44319_2024_179_MOESM10_ESM.zip › Fig.5/Fig.5C/Microscopy/NFkB/GTKO 0.1nM OVA_BF.png]

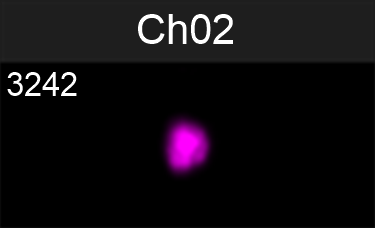

Supplement: Supplementary file 10 — Source data Fig. 5 [file 44319_2024_179_MOESM10_ESM.zip › Fig.5/Fig.5C/Microscopy/NFkB/GTKO 0.1nM OVA_Ch02.png]

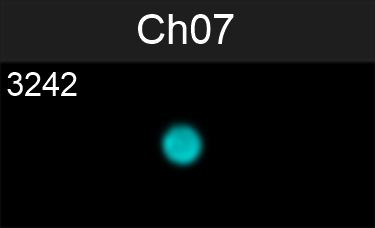

Supplement: Supplementary file 10 — Source data Fig. 5 [file 44319_2024_179_MOESM10_ESM.zip › Fig.5/Fig.5C/Microscopy/NFkB/GTKO 0.1nM OVA_Ch07.png]

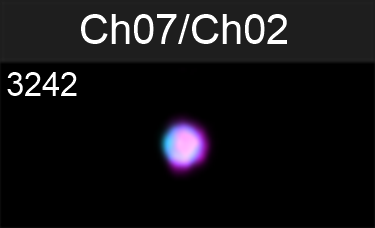

Supplement: Supplementary file 10 — Source data Fig. 5 [file 44319_2024_179_MOESM10_ESM.zip › Fig.5/Fig.5C/Microscopy/NFkB/GTKO 0.1nM OVA_overlay.png]

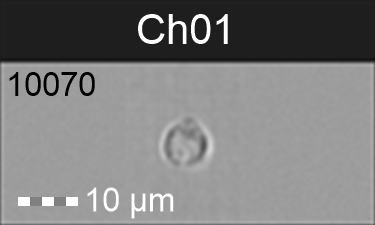

Supplement: Supplementary file 10 — Source data Fig. 5 [file 44319_2024_179_MOESM10_ESM.zip › Fig.5/Fig.5C/Microscopy/NFkB/GTKO 1uM OVA_BF.png]

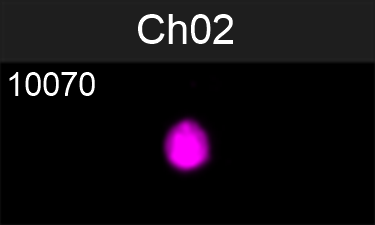

Supplement: Supplementary file 10 — Source data Fig. 5 [file 44319_2024_179_MOESM10_ESM.zip › Fig.5/Fig.5C/Microscopy/NFkB/GTKO 1uM OVA_Ch02.png]

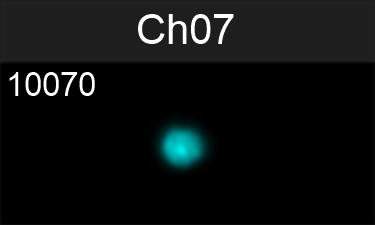

Supplement: Supplementary file 10 — Source data Fig. 5 [file 44319_2024_179_MOESM10_ESM.zip › Fig.5/Fig.5C/Microscopy/NFkB/GTKO 1uM OVA_Ch07.png]

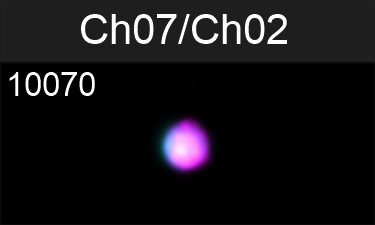

Supplement: Supplementary file 10 — Source data Fig. 5 [file 44319_2024_179_MOESM10_ESM.zip › Fig.5/Fig.5C/Microscopy/NFkB/GTKO 1uM OVA_overlay.png]

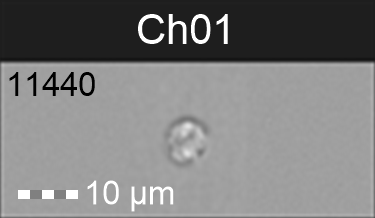

Supplement: Supplementary file 10 — Source data Fig. 5 [file 44319_2024_179_MOESM10_ESM.zip › Fig.5/Fig.5C/Microscopy/NFkB/GTKO no OVA_BF.png]

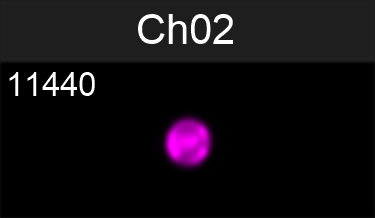

Supplement: Supplementary file 10 — Source data Fig. 5 [file 44319_2024_179_MOESM10_ESM.zip › Fig.5/Fig.5C/Microscopy/NFkB/GTKO no OVA_Ch02.png]

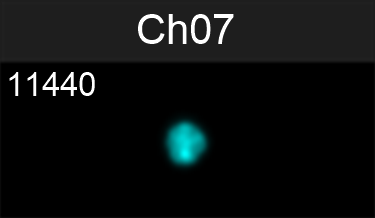

Supplement: Supplementary file 10 — Source data Fig. 5 [file 44319_2024_179_MOESM10_ESM.zip › Fig.5/Fig.5C/Microscopy/NFkB/GTKO no OVA_Ch07.png]

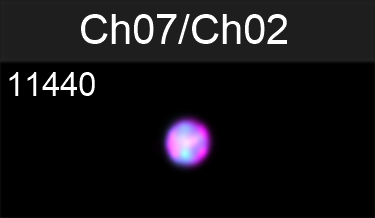

Supplement: Supplementary file 10 — Source data Fig. 5 [file 44319_2024_179_MOESM10_ESM.zip › Fig.5/Fig.5C/Microscopy/NFkB/GTKO no OVA_overlay.png]

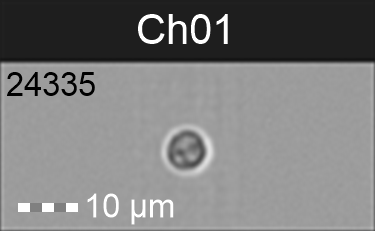

Supplement: Supplementary file 10 — Source data Fig. 5 [file 44319_2024_179_MOESM10_ESM.zip › Fig.5/Fig.5C/Microscopy/NFkB/WT 0.1nM OVA_BF.png]

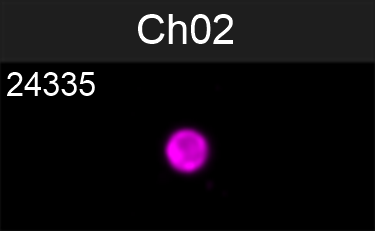

Supplement: Supplementary file 10 — Source data Fig. 5 [file 44319_2024_179_MOESM10_ESM.zip › Fig.5/Fig.5C/Microscopy/NFkB/WT 0.1nM OVA_Ch02.png]

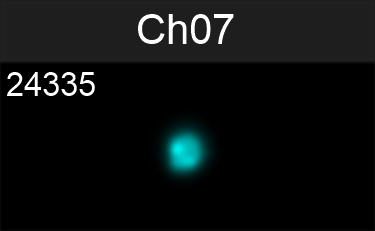

Supplement: Supplementary file 10 — Source data Fig. 5 [file 44319_2024_179_MOESM10_ESM.zip › Fig.5/Fig.5C/Microscopy/NFkB/WT 0.1nM OVA_Ch07.png]

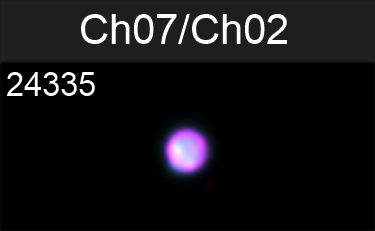

Supplement: Supplementary file 10 — Source data Fig. 5 [file 44319_2024_179_MOESM10_ESM.zip › Fig.5/Fig.5C/Microscopy/NFkB/WT 0.1nM OVA_overlay.png]

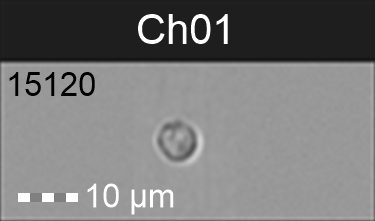

Supplement: Supplementary file 10 — Source data Fig. 5 [file 44319_2024_179_MOESM10_ESM.zip › Fig.5/Fig.5C/Microscopy/NFkB/WT 1uM OVA_BF.png]

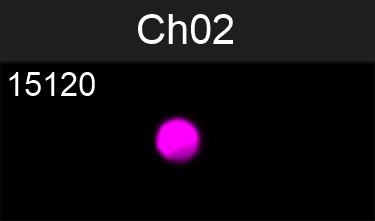

Supplement: Supplementary file 10 — Source data Fig. 5 [file 44319_2024_179_MOESM10_ESM.zip › Fig.5/Fig.5C/Microscopy/NFkB/WT 1uM OVA_Ch02.png]

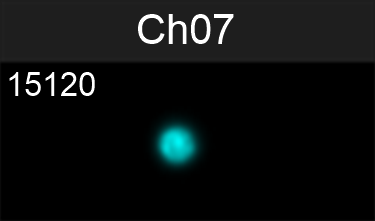

Supplement: Supplementary file 10 — Source data Fig. 5 [file 44319_2024_179_MOESM10_ESM.zip › Fig.5/Fig.5C/Microscopy/NFkB/WT 1uM OVA_Ch07.png]

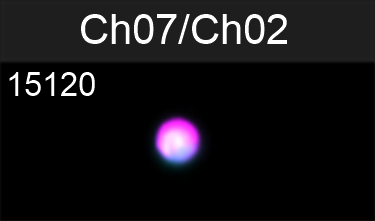

Supplement: Supplementary file 10 — Source data Fig. 5 [file 44319_2024_179_MOESM10_ESM.zip › Fig.5/Fig.5C/Microscopy/NFkB/WT 1uM OVA_overlay.png]

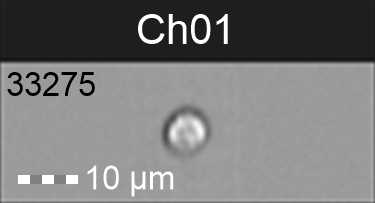

Supplement: Supplementary file 10 — Source data Fig. 5 [file 44319_2024_179_MOESM10_ESM.zip › Fig.5/Fig.5C/Microscopy/NFkB/WT no OVA_BF.png]

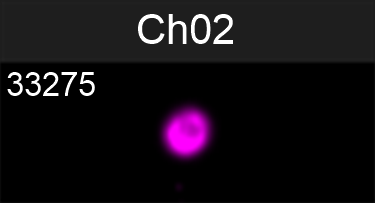

Supplement: Supplementary file 10 — Source data Fig. 5 [file 44319_2024_179_MOESM10_ESM.zip › Fig.5/Fig.5C/Microscopy/NFkB/WT no OVA_Ch02.png]

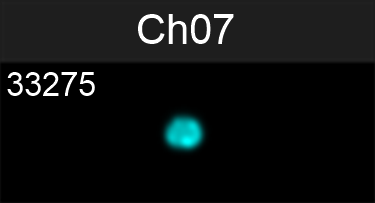

Supplement: Supplementary file 10 — Source data Fig. 5 [file 44319_2024_179_MOESM10_ESM.zip › Fig.5/Fig.5C/Microscopy/NFkB/WT no OVA_Ch07.png]

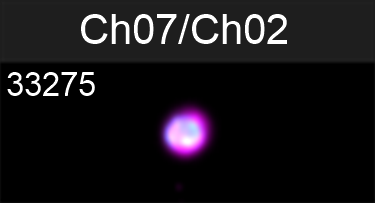

Supplement: Supplementary file 10 — Source data Fig. 5 [file 44319_2024_179_MOESM10_ESM.zip › Fig.5/Fig.5C/Microscopy/NFkB/WT no OVA_overlay.png]

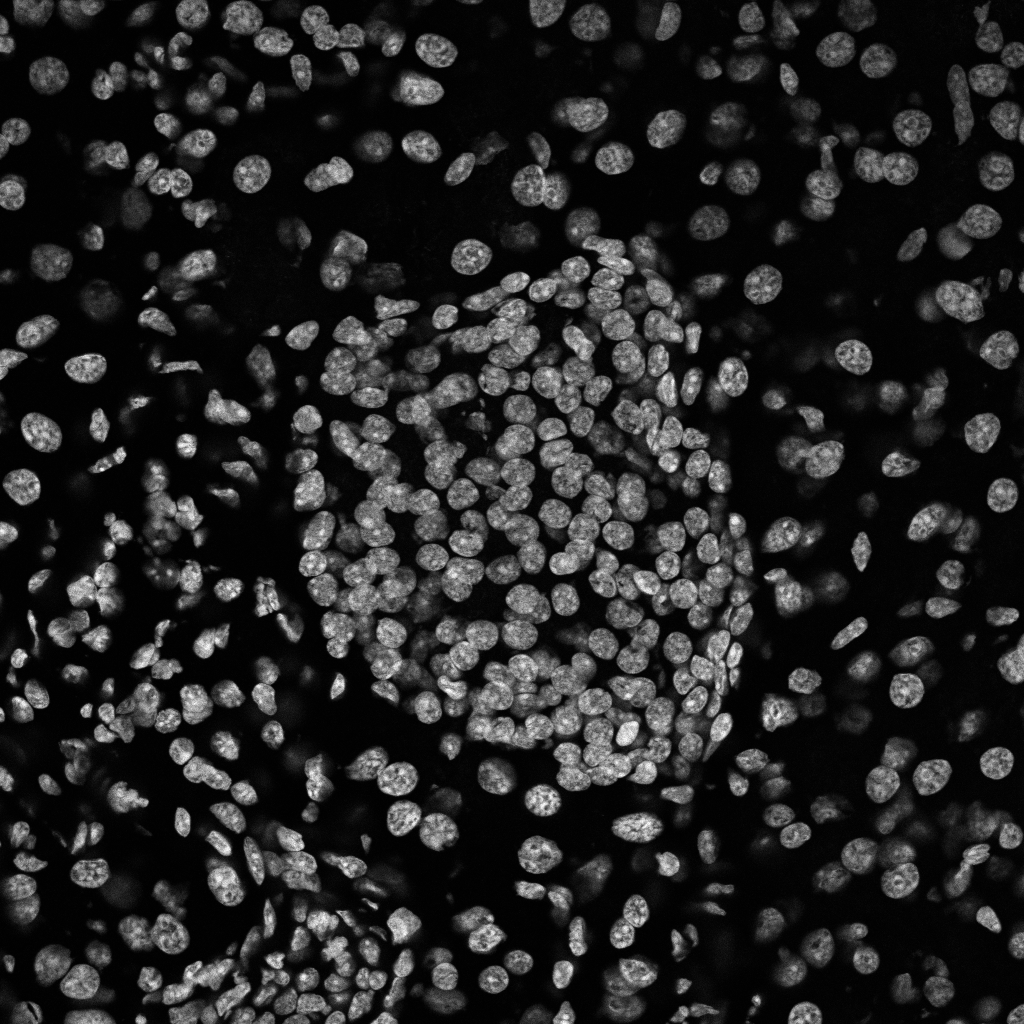

Supplement: Supplementary file 11 — Source data Fig. 6 [file 44319_2024_179_MOESM11_ESM.zip › Fig.6/Fig.6I/Microscopy/CTRL_Ch1_DAPI.tif]

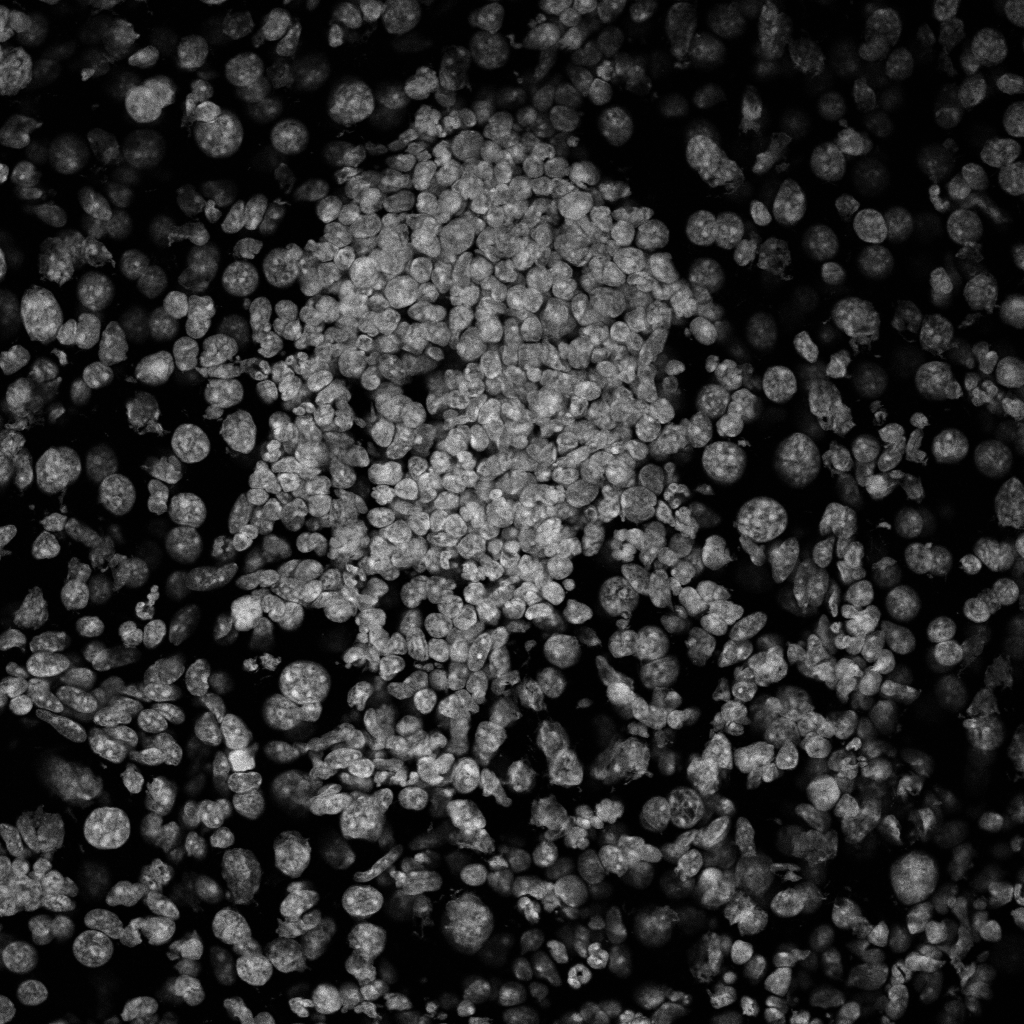

Supplement: Supplementary file 11 — Source data Fig. 6 [file 44319_2024_179_MOESM11_ESM.zip › Fig.6/Fig.6I/Microscopy/GTKO 1000_Ch1_DAPI.tif]

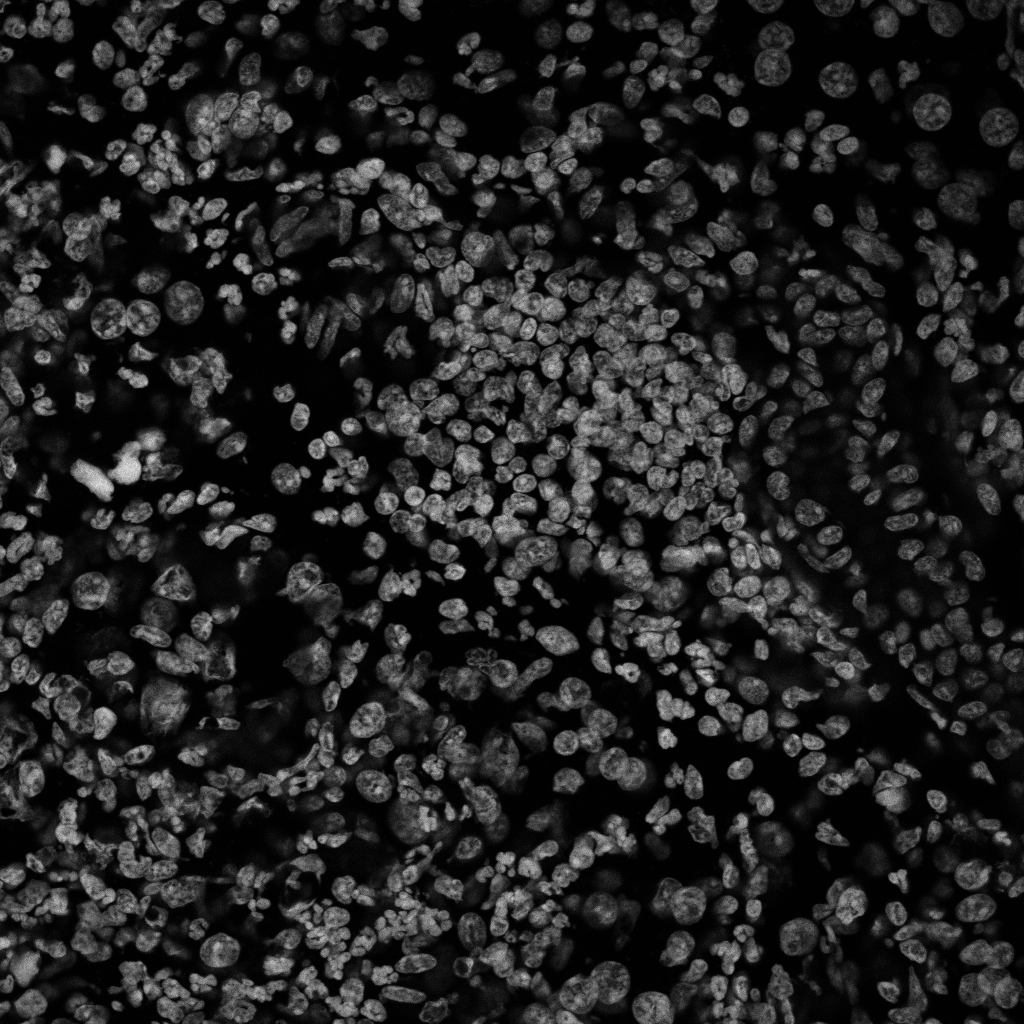

Supplement: Supplementary file 11 — Source data Fig. 6 [file 44319_2024_179_MOESM11_ESM.zip › Fig.6/Fig.6I/Microscopy/GTKO 250_Ch1_DAPI.tif]

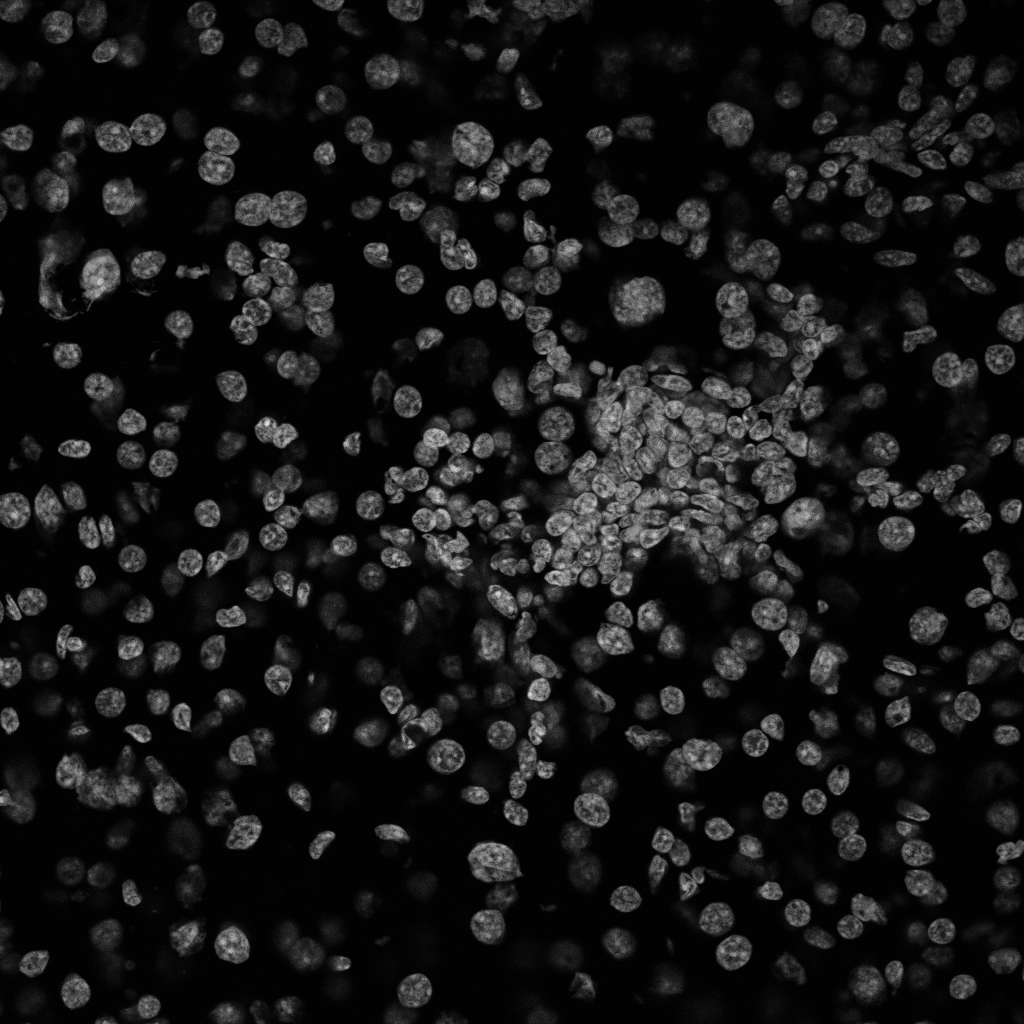

Supplement: Supplementary file 11 — Source data Fig. 6 [file 44319_2024_179_MOESM11_ESM.zip › Fig.6/Fig.6I/Microscopy/WT 1000_Ch1_DAPI.tif]

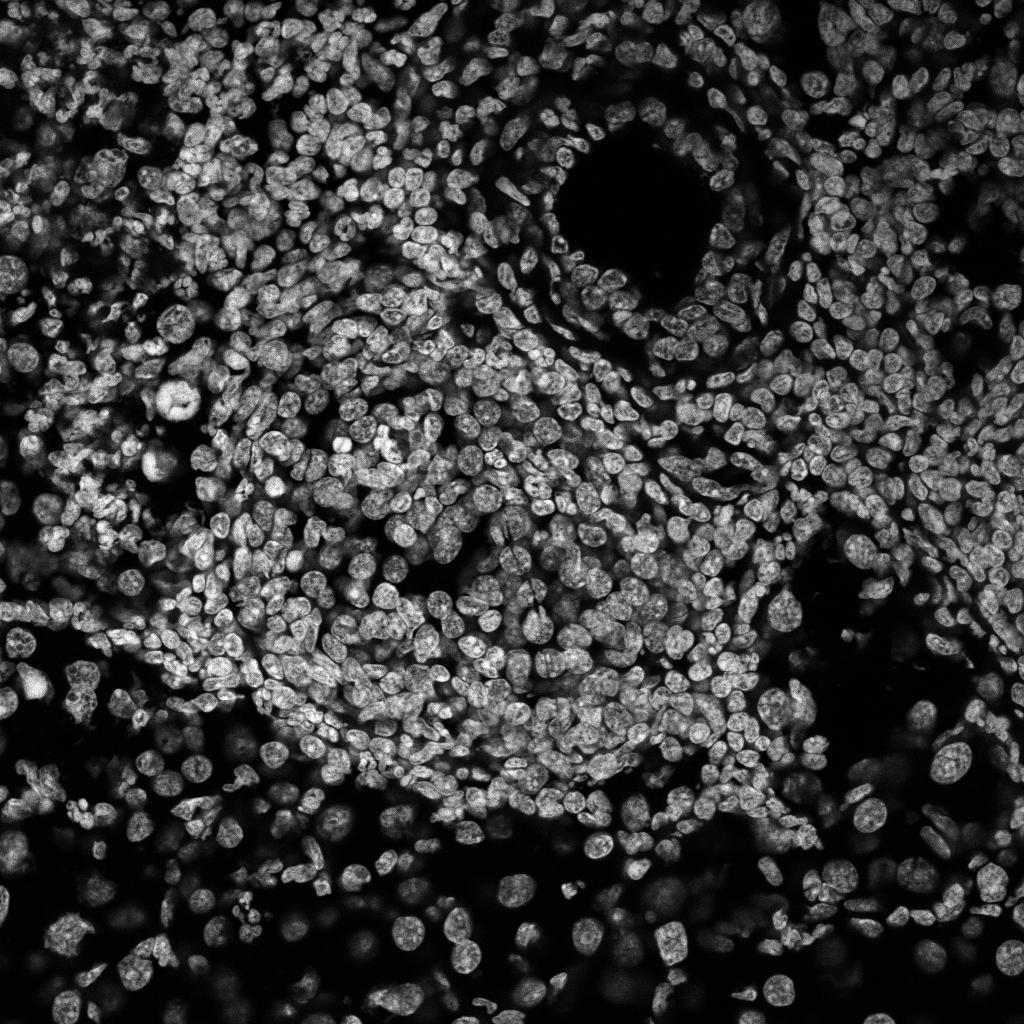

Supplement: Supplementary file 11 — Source data Fig. 6 [file 44319_2024_179_MOESM11_ESM.zip › Fig.6/Fig.6I/Microscopy/WT 250_Ch1_DAPI.tif]
