# Supplementary material for: ABIN1 is a negative regulator of effector functions in cytotoxic T cells
Source: EMBO Rep. 2024 Jun 14;25(8):17. doi: 10.1038/s44319-024-00179-6 (PMC11315980; doi:10.1038/s44319-024-00179-6)
Supplement: Supplementary file 12 — Expanded View Figures [file 44319_2024_179_MOESM12_ESM.pdf]

Expanded View Figures

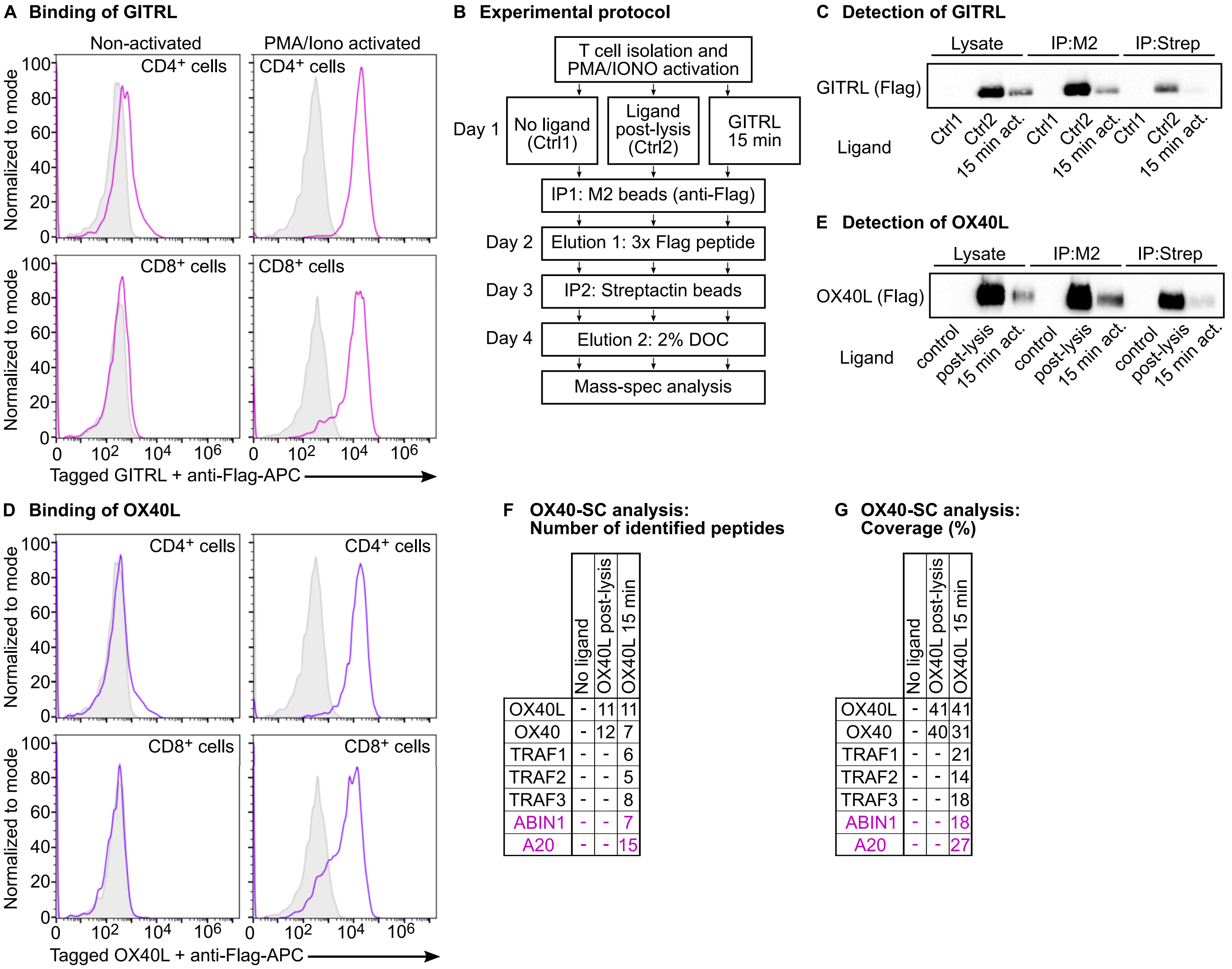

F OX40-SC analysis: Number of identified peptides

	No ligand	OX40L post-lysis	OX40L 15 min
OX40L	-	11	11
OX40	-	12	7
TRAF1	-	-	6
TRAF2	-	-	5
TRAF3	-	-	8
ABIN1	-	-	7
A20	-	-	15

G OX40-SC analysis: Coverage (%)

	No ligand	OX40L post-lysis	OX40L 15 min
OX40L	-	41	41
OX40	-	40	31
TRAF1	-	-	21
TRAF2	-	-	14
TRAF3	-	-	18
ABIN1	-	-	18
A20	-	-	27

Figure EV1. Analysis of the proximal GITR and OX40 signaling complex (SC).

(**A**) Primary murine T cells were pre-activated with PMA/ionomycin for 72 h or not and stained with the anti-CD4 and anti-CD8 antibodies, recombinant GITRL followed by anti-FLAG antibody. A representative experiment out of 3 biological replicates in total. (**B**) Illustration of the protocol for the identification of the composition of GITR and OX40 signaling complexes. (**C**) Detection of the recombinant ligand in the lysate and after the first and second affinity purification step by immunoblotting. A representative experiment out of 3 biological replicates in total. (**D**) Primary murine T cells were pre-activated with PMA/ionomycin for 72 h or not and stained with the anti-CD4 and anti-CD8 antibodies, recombinant OX40 followed by anti-FLAG antibody. A representative experiment out of 3 biological replicates in total. (**E–G**) Primary murine T cells were pre-activated with PMA/ionomycin for 72 h and stimulated with the recombinant OX40L for 15 min. The cells were lysed and the OX40-SC was isolated via tandem affinity purification and samples were analyzed by mass spectrometry. (**E**) Detection of the recombinant ligand in the lysate and after the first and second affinity purification step by immunoblotting. Results of the protein identification are shown as the number of peptides (**F**) and the coverage (**G**). A single experiment was performed.

© The Author(s)

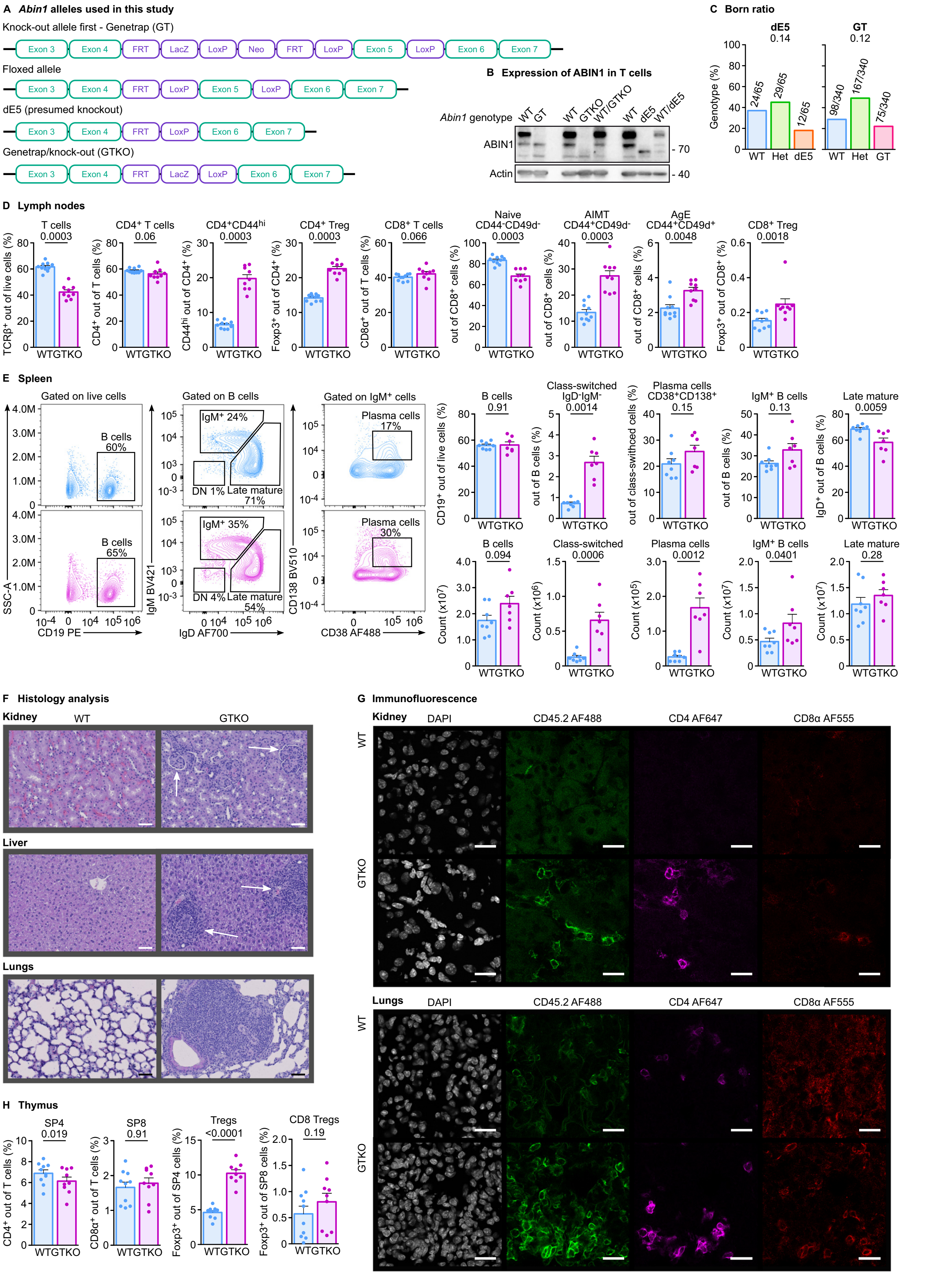
A Abin1 alleles used in this study
Knock-out allele first - Genetrap (GT)
Exon 3 Exon 4 FRT LacZ LoxP Neo FRT LoxP Exon 5 LoxP Exon 6 Exon 7
Floxed allele
Exon 3 Exon 4 FRT LoxP Exon 5 LoxP Exon 6 Exon 7
dE5 (presumed knockout)
Exon 3 Exon 4 FRT LoxP Exon 6 Exon 7
Genetrap/knock-out (GTKO)
Exon 3 Exon 4 FRT LacZ LoxP Exon 6 Exon 7
B Expression of ABIN1 in T cells
Abin1 genotype WT GT WT GTKO WT/GTKO WT dE5 WT/dE5
ABIN1 - 70
Actin - 40
C Born ratio
dE5 0.14
GT 0.12
Genotype (%)
24/65 29/65 12/65
98/340 167/340 75/340
WT Het dE5 WT Het GT
D Lymph nodes
T cells 0.0003
CD4+ T cells 0.06
CD4+CD44hi 0.0003
CD4+ Treg 0.0003
CD8+ T cells 0.066
Naive CD44-CD49d- 0.0003
AIMT CD44+CD49d- 0.0003
AgE CD44+CD49d+ 0.0048
CD8+ Treg 0.0018
TCRβ+ out of live cells (%)
CD4+ out of T cells (%)
CD44hi out of CD4+ (%)
Foxp3+ out of CD4+ (%)
CD8α+ out of T cells (%)
out of CD8+ cells (%)
Foxp3+ out of CD8+ (%)
WT GTKO
E Spleen
Gated on live cells
Gated on B cells
Gated on IgM+ cells
B cells 60%
B cells 65%
IgM+ 24%
IgM+ 35%
DN 1% Late mature 71%
DN 4% Late mature 54%
Plasma cells 17%
Plasma cells 30%
SSC-A
CD19 PE
IgM BV421
IgD AF700
CD138 BV510
CD38 AF488
B cells 0.91
Class-switched IgD-IgM- 0.0014
Class-switched 0.0006
Plasma cells CD38+CD138+ 0.15
Plasma cells 0.0012
IgM+ B cells 0.13
IgM+ B cells 0.0401
Late mature 0.0059
Late mature 0.28
B cells 0.094
CD19+ out of live cells (%)
out of B cells (%)
out of class-swithced cells (%)
IgD+ out of B cells (%)
Count (x10^7)
Count (x10^6)
Count (x10^5)
F Histology analysis
Kidney WT GTKO
Liver
Lungs
G Immunofluorescence
Kidney DAPI CD45.2 AF488 CD4 AF647 CD8α AF555
Lungs DAPI CD45.2 AF488 CD4 AF647 CD8α AF555
WT
GTKO
H Thymus
SP4 0.019
SP8 0.91
Tregs <0.0001
CD8 Tregs 0.19
CD4+ out of T cells (%)
CD8α+ out of T cells (%)
Foxp3+ out of SP4 cells (%)
Foxp3+ out of SP8 cells (%)

Figure EV2. Characterization of Abin1$^{GT/GT}$ (GT), Abin1$^{dE5/dE5}$ (dE5), and Abin1$^{GTKO/GTKO}$ (GTKO) mice.

(**A**) An overview of *Abin1* alleles used in this study. (**B**) Immunoblot analysis of ABIN1 in GT, GTKO, dE5 mice and corresponding littermate controls. A representative experiment is shown. The experiment was performed in 5 (GT), 3 (GTKO), or 2 (dE5) biological replicates. (**C**) Heterozygous *Abin1*$^{WT/dE5}$ or *Abin1*$^{WT/GT}$, respectively, were bred and the genotype of the offspring was determined upon weaning. The frequencies and numbers of pups with particular genotypes are indicated. $n = 340$ (GT) or 65 (dE5) offspring mice per group in total from 16 (GT) or 2 (dE5) breedings. (**D**) Lymph node cells were stained with indicated antibodies and analyzed by flow cytometry. Aggregate results of the abundance of indicated subsets are shown, $n = 10$ (WT) or 9 (GTKO) mice per group. (**E**) Splenocytes were stained with indicated antibodies and analyzed by flow cytometry. Representative dot plots and aggregate results of the frequency of indicated subsets are shown. $n = 10$ (WT) or 9 (GTKO) mice per group. (**F**) Histological analysis using H&E staining of indicated organs of 20–26 week-old mice. Arrows pointing to inflammatory foci. Representative staining out of 4 mice per group in total. (**G**) Cryosections of lungs and kidneys of WT and GTKO mice were stained with indicated antibodies and DAPI (nuclei) and analyzed by confocal fluorescence microscopy. Representative sections out of 4 mice per group in total. (**H**) Fixed and permeabilized thymocytes from WT and GTKO mice were stained with indicated antibodies and analyzed by flow cytometry. $n = 10$ (WT) or 9 (GTKO) mice per group. Data information: In (**D**, **E**, **H**), data are represented as mean + SEM and *p* values are indicated. In (**F**, **G**), the scale bars indicate 20 μm and 50 μm, respectively. Statistical significance was determined by a binomial test (**A**) or two-tailed Mann–Whitney test (**D**, **E**, **H**).

 © The Author(s)

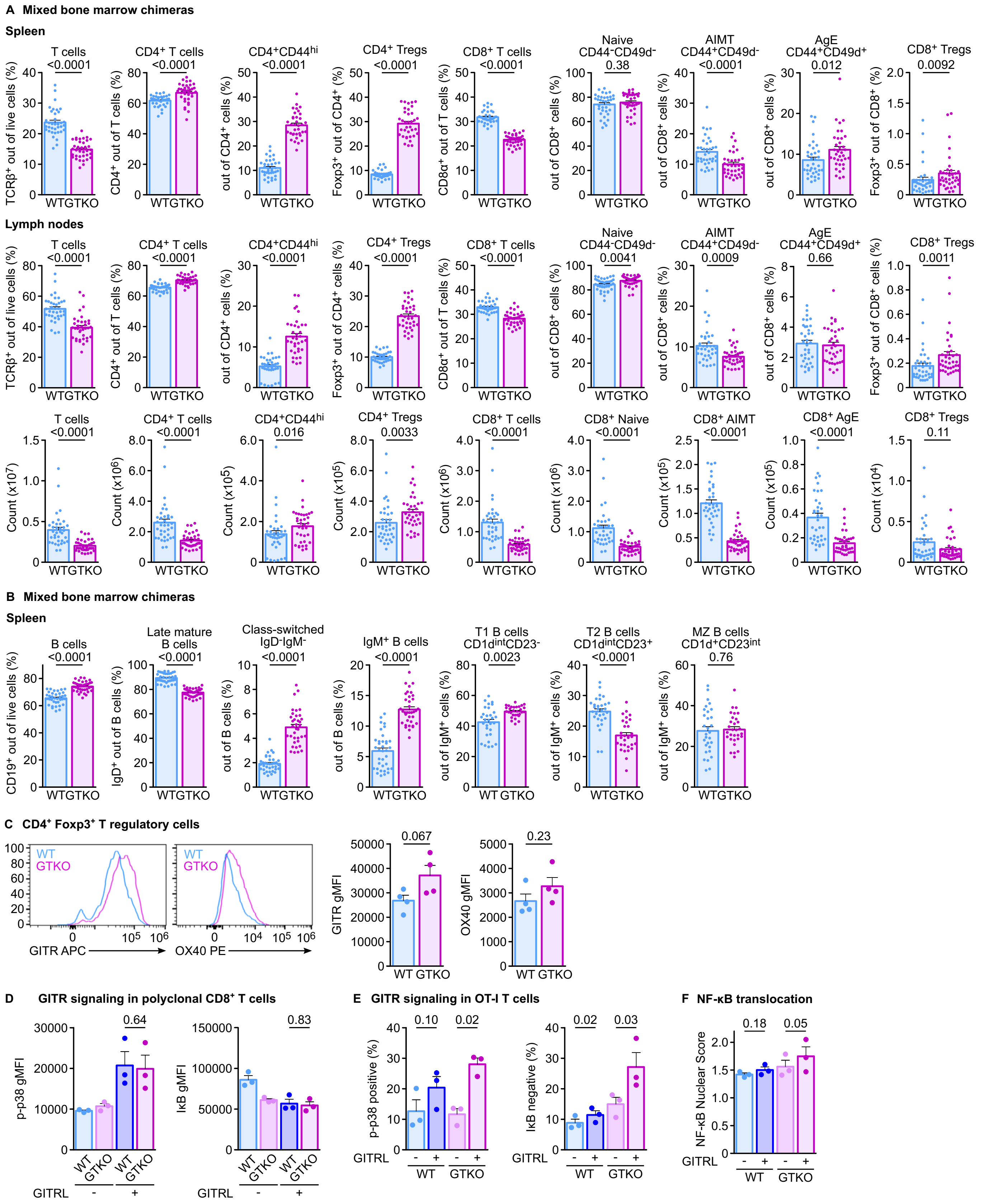
A Mixed bone marrow chimeras
Spleen
T cells <0.0001
CD4+ T cells <0.0001
CD4+CD44hi <0.0001
CD4+ Tregs <0.0001
CD8+ T cells <0.0001
Naive CD44-CD49d- 0.38
AIMT CD44+CD49d- <0.0001
AgE CD44+CD49d+ 0.012
CD8+ Tregs 0.0092
TCRβ+ out of live cells (%)
CD4+ out of T cells (%)
out of CD4+ cells (%)
Foxp3+ out of CD4+ (%)
CD8α+ out of T cells (%)
out of CD8+ cells (%)
Foxp3+ out of CD8+ (%)
WT GTKO
Lymph nodes
T cells <0.0001
CD4+ T cells <0.0001
CD4+CD44hi <0.0001
CD4+ Tregs <0.0001
CD8+ T cells <0.0001
Naive CD44-CD49d- 0.0041
AIMT CD44+CD49d- 0.0009
AgE CD44+CD49d+ 0.66
CD8+ Tregs 0.0011
T cells <0.0001
CD4+ T cells <0.0001
CD4+CD44hi 0.016
CD4+ Tregs 0.0033
CD8+ T cells <0.0001
CD8+ Naive <0.0001
CD8+ AIMT <0.0001
CD8+ AgE <0.0001
CD8+ Tregs 0.11
Count (x10^7)
Count (x10^6)
Count (x10^5)
Count (x10^4)
B Mixed bone marrow chimeras
Spleen
B cells <0.0001
Late mature B cells <0.0001
Class-switched IgD-IgM- <0.0001
IgM+ B cells <0.0001
T1 B cells CD1dintCD23- 0.0023
T2 B cells CD1dintCD23+ <0.0001
MZ B cells CD1d+CD23int 0.76
CD19+ out of live cells (%)
IgD+ out of B cells (%)
out of B cells (%)
out of IgM+ cells (%)
C CD4+ Foxp3+ T regulatory cells
WT
GTKO
GITR APC
OX40 PE
GITR gMFI 0.067
OX40 gMFI 0.23
D GITR signaling in polyclonal CD8+ T cells
p-p38 gMFI 0.64
IκB gMFI 0.83
GITRL - +
E GITR signaling in OT-I T cells
p-p38 positive (%) 0.10 0.02
IκB negative (%) 0.02 0.03
GITRL - + - +
WT GTKO
F NF-κB translocation
NF-κB Nuclear Score 0.18 0.05

Figure EV3. Intrinsic roles of ABIN1 in T cells.

(**A**, **B**) The experiment shown in Fig. 3A,B. Aggregate results of the frequency and absolute counts of indicated subsets of T cells (**A**) or B cells (**B**). $n = 36$ mice per group. (**C**) Lymph node cells from WT or *Abin1*$^{GTKO/GTKO}$ mice were analyzed by flow cytometry. The expression of GITR and OX40 on $CD4^+$ $FOXP3^+$ T cells is shown. Representative histograms and aggregate data are shown. $n = 4$ mice per group. (**D**) The experiment shown in Fig. 3D. Lymph node cells from WT or *Abin1*$^{GTKO/GTKO}$ mice were pre-activated with PMA/ionomycin and stimulated with GITRL or left untreated (controls). Indicated activation pathways were analyzed by flow cytometry. Aggregate results of phospho-p38 and IκB levels in $CD8^+$ T cells. $n = 4$ mice per group. (**E**, **F**) The experiment shown in Fig. 3E. Lymph node cells from WT or *Abin1*$^{GTKO/GTKO}$ OT-I *Rag2*$^{KO/KO}$ mice were pre-activated with PMA/ionomycin and stimulated with GITRL or left untreated and analyzed by flow cytometry (**E**) or flow imaging (**F**). $n = 3$ mice per group. (**E**) The Percentage of phospho-p38 and IκB positive cells is shown. (**F**) The nuclear translocation score for NF-κB is shown. Data information: In (**A**–**E**), the data are presented as mean + SEM and *P* values are indicated. Statistical significance was determined by two-tailed Mann–Whitney test (**A**, **B**) or two-tailed Student's *t* test (**C**, **D**) or one-tailed paired *t* test (**E**, **F**).

 © The Author(s)

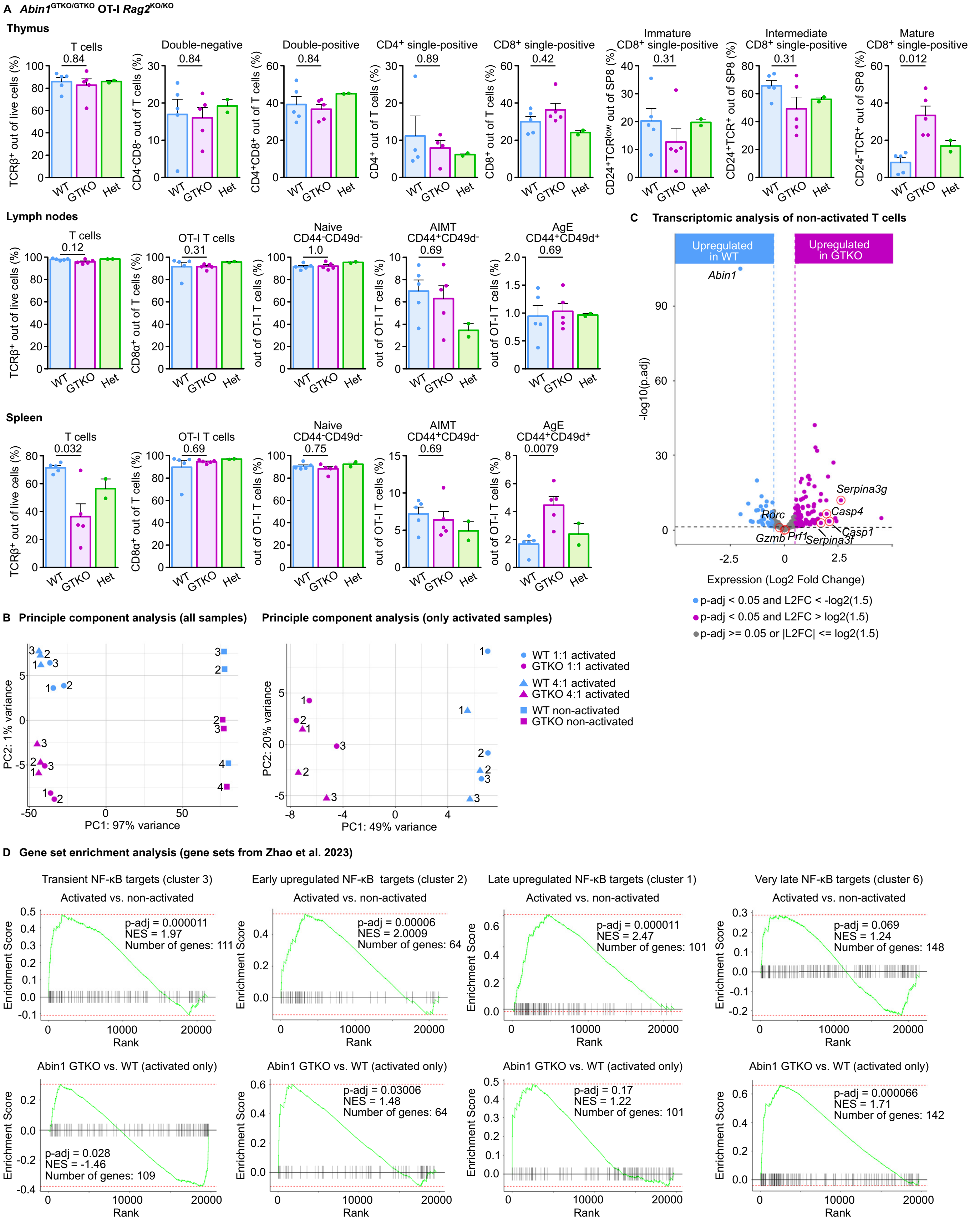
A Abin1 GTKO/GTKO OT-I Rag2 KO/KO
Thymus
T cells 0.84
Double-negative 0.84
Double-positive 0.84
CD4+ single-positive 0.89
CD8+ single-positive 0.42
Immature CD8+ single-positive 0.31
Intermediate CD8+ single-positive 0.31
Mature CD8+ single-positive 0.012
Lymph nodes
T cells 0.12
OT-I T cells 0.31
Naive CD44-CD49d- 1.0
AIMT CD44+CD49d- 0.69
AgE CD44+CD49d+ 0.69
Spleen
T cells 0.032
OT-I T cells 0.69
Naive CD44-CD49d- 0.75
AIMT CD44+CD49d- 0.69
AgE CD44+CD49d+ 0.0079
WT GTKO Het
C Transcriptomic analysis of non-activated T cells
Upregulated in WT
Upregulated in GTKO
Abin1
Serpina3g
Casp4
Rorc
Casp1
Gzmb Prf1 Serpina3f
-log10(p.adj)
Expression (Log2 Fold Change)
p-adj < 0.05 and L2FC < -log2(1.5)
p-adj < 0.05 and L2FC > log2(1.5)
p-adj >= 0.05 or |L2FC| <= log2(1.5)
B Principle component analysis (all samples)
Principle component analysis (only activated samples)
PC1: 97% variance
PC2: 1% variance
PC1: 49% variance
PC2: 20% variance
WT 1:1 activated
GTKO 1:1 activated
WT 4:1 activated
GTKO 4:1 activated
WT non-activated
GTKO non-activated
D Gene set enrichment analysis (gene sets from Zhao et al. 2023)
Transient NF-κB targets (cluster 3)
Early upregulated NF-κB targets (cluster 2)
Late upregulated NF-κB targets (cluster 1)
Very late NF-κB targets (cluster 6)
Activated vs. non-activated
p-adj = 0.000011 NES = 1.97 Number of genes: 111
p-adj = 0.00006 NES = 2.0009 Number of genes: 64
p-adj = 0.000011 NES = 2.47 Number of genes: 101
p-adj = 0.069 NES = 1.24 Number of genes: 148
Abin1 GTKO vs. WT (activated only)
p-adj = 0.028 NES = -1.46 Number of genes: 109
p-adj = 0.03006 NES = 1.48 Number of genes: 64
p-adj = 0.17 NES = 1.22 Number of genes: 101
p-adj = 0.000066 NES = 1.71 Number of genes: 142
Enrichment Score
Rank

Figure EV4. Characterization of *Abin1*$^{GTKO/GTKO}$ OT-I mice.

(**A**) Cells from lymph nodes, spleen, and thymus from *Abin1*$^{WT/WT}$ OT-I *Rag2*$^{KO/KO}$ (WT), *Abin1*$^{GTKO/GTKO}$ OT-I *Rag2*$^{KO/KO}$ (GTKO), and *Abin1*$^{WT/GTKO}$ OT-I *Rag2*$^{KO/KO}$ (HET) were stained with indicated antibodies and analyzed by flow cytometry. Aggregate results of the frequency of indicated subsets are shown. $n = 5$ (WT and GTKO) or 2 (HET) mice per group. (**B**) Principal component analysis of the RNAseq experiment shown in Fig. 4C,D. (**C**) A volcano plot showing the differences in gene expression between non-activated WT and GTKO T cells from the experiment shown in Fig. 4C,D. (**D**) Four clusters of NF-κB responsive genes with distinct expression kinetics after the antigenic signaling were taken from a study by Zhao et al (Zhao et al, 2023). Using these gene lists, we performed a gene set enrichment analysis for the contrast between activated vs. non-activated OT-I T cells (upper lane) and activated GTKO vs. activated WT OT-I T cells (bottom lane) using the RNAseq data shown in Fig. 4C,D. Data information: In (**A**), the data are presented as mean + SEM. The statistical significance was calculated using two-tailed Mann–Whitney test (**A**), Wald test with Benjamini–Hochberg multiple testing correction (**C**), or Weighted Kolmogorov–Smirnov test (**D**).

 © The Author(s)

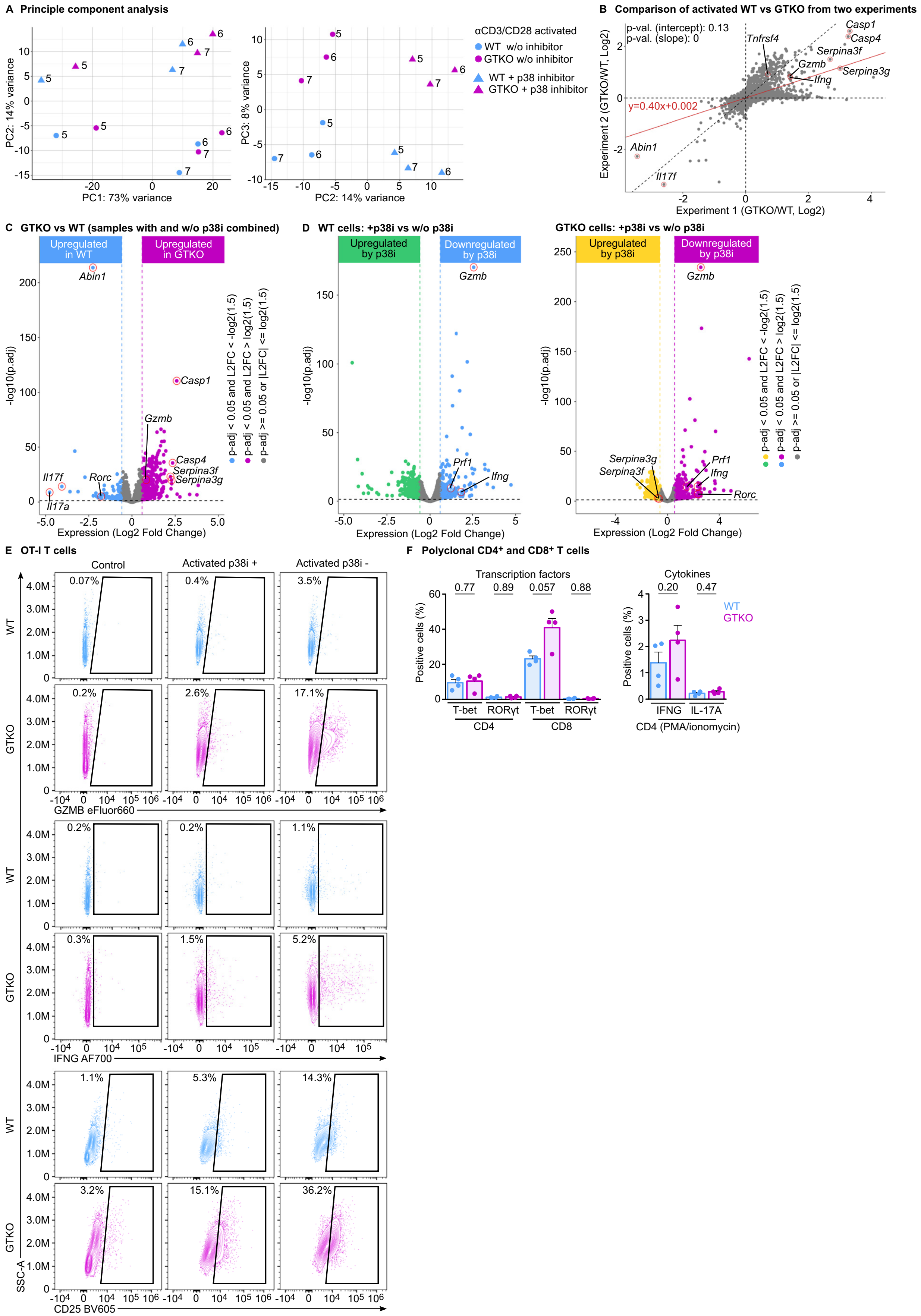
A Principle component analysis
αCD3/CD28 activated
WT w/o inhibitor
GTKO w/o inhibitor
WT + p38 inhibitor
GTKO + p38 inhibitor
PC1: 73% variance
PC2: 14% variance
PC3: 8% variance
B Comparison of activated WT vs GTKO from two experiments
p-val. (intercept): 0.13
p-val. (slope): 0
y=0.40x+0.002
Experiment 1 (GTKO/WT, Log2)
Experiment 2 (GTKO/WT, Log2)
C GTKO vs WT (samples with and w/o p38i combined)
Upregulated in WT
Upregulated in GTKO
D WT cells: +p38i vs w/o p38i
Upregulated by p38i
Downregulated by p38i
GTKO cells: +p38i vs w/o p38i
-log10(p.adj)
Expression (Log2 Fold Change)
p-adj < 0.05 and L2FC < -log2(1.5)
p-adj < 0.05 and L2FC > log2(1.5)
p-adj >= 0.05 or |L2FC| <= log2(1.5)
E OT-I T cells
Control
Activated p38i +
Activated p38i -
GZMB eFluor660
IFNG AF700
CD25 BV605
SSC-A
F Polyclonal CD4+ and CD8+ T cells
Transcription factors
Cytokines
Positive cells (%)
T-bet
RORγt
CD4
CD8
IFNG
IL-17A
CD4 (PMA/ionomycin)
WT
GTKO

Figure EV5. Analysis of signaling pathways in ABIN1-deficient T cells.

(**A–D**) The same RNAseq experiment as presented in Fig. 5E,F. $n = 3$ biological replicates. (**A**) Principal component analysis of the samples. (**B**) Comparison of these experiments and the previous set of experiments (presented in Fig. 4C,D) by plotting the fold changes of activated *Abin1*$^{GTKO/GTKO}$ OT-I *Rag2*$^{KO/KO}$ (GTKO) vs. *Abin1*$^{WT/WT}$ OT-I *Rag2*$^{KO/KO}$ (WT) T cells in both sets of experiments. (**C**) A volcano plot showing expression changes of activated T cells treated p38 MAPK inhibitor (12.5 μM) for combined WT and GTKO samples. (**D**) A volcano plot showing expression changes of activated T cells treated or non-treated with p38 MAPK inhibitor (12.5 μM) for WT and GTKO samples separately. (**E**) Lymph node cells from WT and GTKO OT-I mice from the experiment shown in Fig. 5G. Representative dot plots showing the expression of indicated markers are shown. $n = 7$ (WT), or 8 (GTKO) mice per group. (**F**) Lymph node cells from WT or GTKO polyclonal mice were analyzed by flow cytometry for the expression of indicated transcription factors (left) or activated with PMA/ionomycin for 4 h and analyzed by flow cytometry for the production of indicated cytokines (right). $n = 4$ mice per group. Data information: In (**F**), data are presented as mean + SEM. The statistical analysis was calculated using two-tailed Wald test with Benjamini–Hochberg multiple testing correction (**C**, **D**) or two-tailed Student's *t* test (**F**).

 © The Author(s)

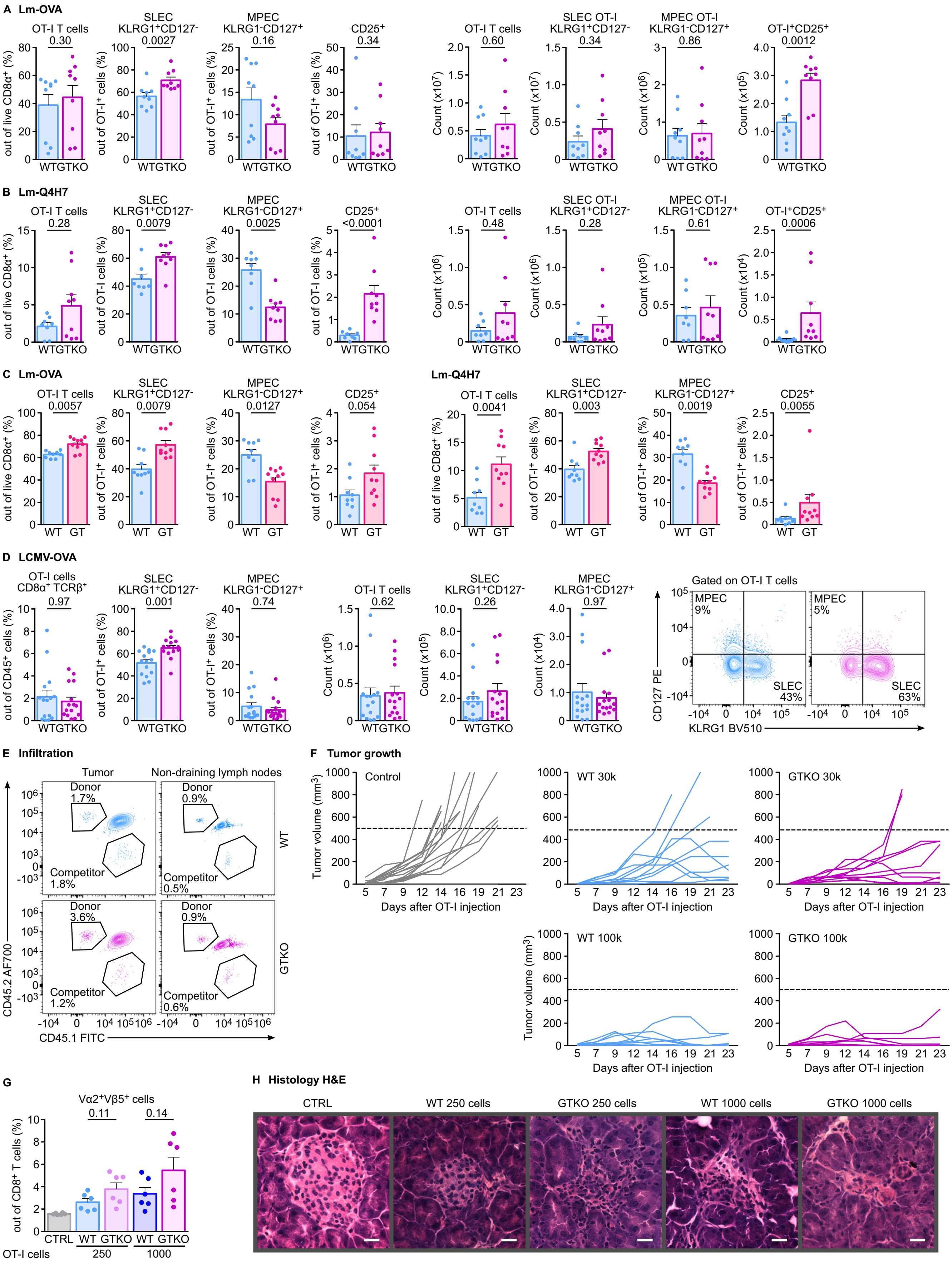
A Lm-OVA
OT-I T cells
SLEC KLRG1+CD127-
MPEC KLRG1-CD127+
CD25+
SLEC OT-I
MPEC OT-I
OT-I+CD25+
0.30
0.0027
0.16
0.34
0.60
0.34
0.86
0.0012
out of live CD8α+ (%)
out of OT-I+ cells (%)
Count (x10^7)
Count (x10^6)
Count (x10^5)
WT GTKO
B Lm-Q4H7
0.28
0.0079
0.0025
<0.0001
0.48
0.28
0.61
0.0006
Count (x10^4)
C Lm-OVA
Lm-Q4H7
0.0057
0.0079
0.0127
0.054
0.0041
0.003
0.0019
0.0055
WT GT
D LCMV-OVA
OT-I cells CD8α+ TCRβ+
0.97
0.001
0.74
0.62
0.26
0.97
out of CD45+ cells (%)
Gated on OT-I T cells
MPEC 9%
SLEC 43%
MPEC 5%
SLEC 63%
CD127 PE
KLRG1 BV510
E Infiltration
Tumor
Non-draining lymph nodes
Donor 1.7%
Competitor 1.8%
Donor 0.9%
Competitor 0.5%
Donor 3.6%
Competitor 1.2%
Donor 0.9%
Competitor 0.6%
WT
GTKO
CD45.2 AF700
CD45.1 FITC
F Tumor growth
Control
WT 30k
GTKO 30k
WT 100k
GTKO 100k
Tumor volume (mm3)
Days after OT-I injection
G
Vα2+Vβ5+ cells
0.11
0.14
out of CD8+ T cells (%)
CTRL WT GTKO WT GTKO
OT-I cells 250 1000
H Histology H&E
CTRL
WT 250 cells
GTKO 250 cells
WT 1000 cells
GTKO 1000 cells

© The Author(s)

Figure EV6. ABIN1 regulates T-cell responses in vivo.

(**A**, **B**) The experiments as shown in Fig. 6A,B with identical protocol, but using a transfer of 5×10^4 T cells. (**A**) $n = 9$ mice per group. (**B**) $n = 8$ (WT), or 9 (GTKO) mice per group. Quantified frequencies and counts of indicated subsets of donor cells are shown. (**C**) 1×10^5 OT-I cells from WT or *Abin1*$^{GT/GT}$ OT-I *Rag2*$^{KO/KO}$ (GT) mice were adoptively transferred to Ly5.2 hosts that were infected with Lm-OVA. Splenocytes were analyzed by flow cytometry on day 6 post infection. The frequency of indicated subsets is shown. $n = 9$ (WT) or 10 (GT) mice per group. (**D**) 1×10^4 OT-I cells from WT or *Abin1*$^{GTKO/GTKO}$ OT-I *Rag2*$^{KO/KO}$ mice were adoptively transferred to the *CD3ε*$^{KO/KO}$ hosts that were infected with LCMV expressing OVA peptide. Splenocytes were analyzed by flow cytometry on day 5 post infection. Representative dot plots and quantified frequencies and absolute counts of indicated subsets of donor cells are shown. $n = 15$ mice per group. (**E**) The experiment shown in Fig. 6C. A representative experiment for tumor and non-draining lymph nodes. (**F**) The experiment shown in Fig. 6D. The tumor growth in individual mice is shown. The dashed line represents the endpoint of the experiment (tumor volume 500 mm^3). The genotype and number of transferred OT-I *Rag2*$^{KO/KO}$ T cells are indicated. (**G**) In the experimental autoimmune assay (Fig. 6F), splenocytes of the host mice were analyzed by flow cytometry on day 6. The frequency of $V\alpha2^+$ $V\beta5^+$ cells among all $CD8^+$ T cells was quantified as a proxy for donor OT-I T cells. $n = 6$ mice per group. (**H**) Pancreatic cryosections (same experiments as shown in Fig. 6I) were stained with hematoxylin and eosin and imaged by microscopy. Data information: In (**A–D**, **G**), data are presented as mean + SEM. In (**H**), the scale bars represent 20 μm. The statistical significance was calculated using two-tailed Mann–Whitney test (**A–D**) or two-tailed Student's *t* test (**G**).

© The Author(s)
